# Lipophilic Cations as Mitochondria-Targeting Moieties: Recent Progress and Design Principles for Medicinal Chemistry

**DOI:** 10.1021/acs.jmedchem.5c02076

**Published:** 2025-11-17

**Authors:** Ivan Džajić, Tihomir Tomašič, Luis A. Pardo, Lucija Peterlin Mašič, Andrej Emanuel Cotman

**Affiliations:** † Faculty of Pharmacy, 63721 University of Ljubljana, Aškerčeva cesta 7, 1000 Ljubljana, Slovenia; ‡ 28284 Max Planck Institute for Multidisciplinary Sciences, City Campus, Herrmann-Rein-Str. 3, 37075 Göttingen, Germany

## Abstract

Mitochondria-targeting technology, in the form of (lipophilic cation)–(small molecule) conjugates, was first discussed more than 50 years ago. Since then, the triphenylphosphonium (TPP) cation has become synonymous with the concept of a mitochondria-targeting moiety (MTM). The discovery of its ability to accumulate in mitochondria occurred alongside research on mitochondrial functions and the mechanisms underlying cation import. The recognition of intrinsic biological effects of TPP, apart from delivery of functional cargo, came much later, prompting the development of novel MTMs beyond the archetypal TPP. In this Perspective, we present the current understanding of the biological mechanisms of action of mitochondria-targeting conjugates, describe the methods used for their validation, and overview the recently developed novel mitochondria-targeting moieties. Building upon the recent advances, we propose a rational approach for the development of novel MTMs to be incorporated into the future MTM–linker–cargo therapeutics (**MITACs**, **MIT**ochondria-**TA**rgeting **C**onjugate**s**).

## Graphical abstract

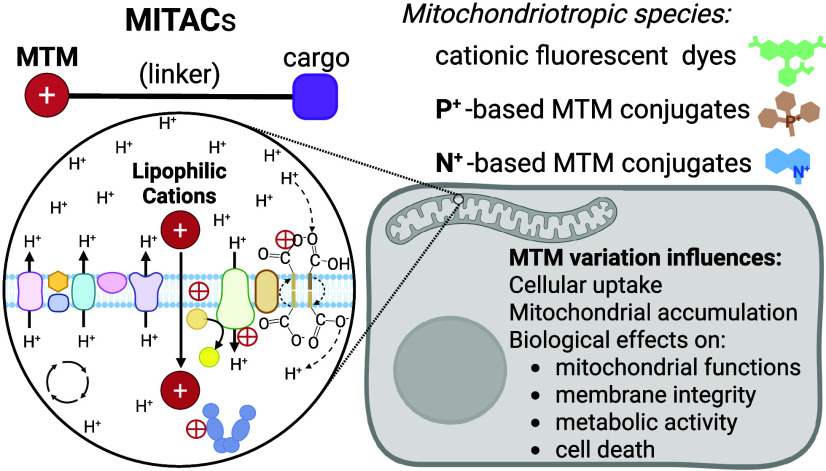

## Significance

This Perspective reviews current approaches to designing mitochondria-targeting conjugates (MITACs) and examines how the structural and physicochemical properties of mitochondria-targeting moieties (MTMs) influence mitochondrial delivery and intrinsic bioactivity. It outlines practical considerations for the experimental evaluation of new MTMs, with the goal of promoting their more consistent profiling and benchmarking in medicinal chemistry research.

## Introduction

Mitochondria-targeted therapy stands out as one of the most promising approaches within organelle-targeted strategies as potential solutions to the unresolved therapeutic challenges.^1–3^ Historically, mitochondria have been primarily regarded as the cellular compartments responsible for generating most of the cell’s energy in the form of ATP, reinforced by their description as *the powerhouse of the cell*.^4^ Their other key roles have been greatly overlooked, but are steadily coming to the forefront. These include (patho)­physiological functions in ROS,^5^ ion,^6,7^ and nucleic acid trafficking,^8,9^ and how these functions relate to cell signaling, metabolism, stress, survival, and death.^3^

Mitochondrial targets have emerged as a central yet challenging focus in oncological drug discovery and beyond.^10–12^ Clinical setbacks with the complex I inhibitor IACS-010759 ^13^ and the lipoic acid analog CPI-613 ^14^ should be regarded not as dead ends but as checkpoints to reassess strategies for targeting mitochondrial function. The recent FDA approvals of an allosteric agonist of the mitochondrial protease ClpP dordaviprone in August 2025,^15^ and of the cardiolipin-binding stabilizer of the inner mitochondrial membrane (IMM) elamipretide in September 2025,^16^ offer strong clinical validation for mitochondrial-targeted therapies. A promising strategy for achieving selective activity within mitochondria (including selective modulation of mitochondrial variants of therapeutic targets), while minimizing effects in other cellular compartments, is by designing the mitochondria-targeting conjugates (MITACs).^17^ These consist of mitochondria-targeting moiety (MTM), linker, and cargo to be delivered to mitochondria. The lipophilic permanent cations have been the most extensively explored as MTMs, including in-human studies.^18–20^ MTMs do more than simply localize functional cargo in mitochondria: depending on the type of MTM and concentration of the conjugate, they induce mitochondrial uncoupling to varying extents,^21^ leading to depolarization and induction of cell death,^22^ or they can cause mitochondrial permeabilization that leads to swelling, potentially due to detergent^23^ or prooxidant action.^24^ Considering the computational and experimental observations, some general conclusions can be drawn regarding how conjugate structure, lipophilicity, charge screening, and (de)­localization affect cellular and mitochondrial permeation kinetics, cyto- or mitotoxicity, and uncoupling, although there is still no consensus on the definitive mechanistic relationships among them.^21,23,25–27^ From a medicinal chemist’s standpoint, various MTMs are exchangeable and represent a diversification point in the optimization of pharmacological properties of mitochondria-targeting compounds.^22^

In experimental settings where multiple methods can assess mitochondrial accumulation and treatment-induced effects (e.g., influence on mitochondrial dynamics, cell death, and viability), some methods outperform others depending on the context. As such, there is a preferential order for selecting methods when validating a novel MTM. This Perspective aims to highlight the most effective methods in each case, present the current consensus and understanding regarding MTMs, point to challenges that still require addressing, as well as to serve as another step toward a more rational approach for designing future mitochondria-targeting therapeutics.

A call for more specific and informative terminology in mitochondrial science has been issued, which is equally relevant to the medicinal chemistry community.^28^ This particularly concerns oversimplified or ambiguously used buzzwords, including “mitochondrial function,” “mitochondrial dysfunction,” “cancer cell mitochondrial hyperpolarization,” “delocalized cation,” and “mitotoxicity”. Even when initially well-defined, the meaning of these terms can become blurred, as they are propagated through successive citations. Incorporating a minimal level of experimental specificity, such as specifying the exact cancer cell line, identifying the assay used to measure membrane potential, describing how charge distribution was assessed, defining the target or mechanism underlying mitochondrial toxicity, or distinguishing the contributions of the MTM versus the cargo in a MITAC to the overall bioactivity would substantially improve clarity and facilitate cross-disciplinary communication. Both terminological *and* experimental standardization and harmonization are superior to individual approaches, and yield better future research outcomes, especially now, when the field of mitochondrial research has gathered a critical amount of data points.

## Mitochondrial Membrane Potential Is the Basis for Lipophilic Cation Uptake

During electron transport along the respiratory chain, protons are exported from the mitochondrial matrix into the intermembrane space against the concentration gradient. This way, the mitochondria maintain both a negative electrochemical gradient and a proton gradient across the inner membrane (transmembrane potential, ΔΨm + ΔpH).^29^ The generation of ΔΨm + ΔpH is considered to be the “mother” of many other mitochondrial functions and behaviors.^28^ It serves not only as a source of thermodynamic free energy to generate ATP in electron transport coupled to oxidative phosphorylation,^30^ or heat by proton leak (uncoupling), but for multiple biosynthetic pathways, uptake of ions (Ca^2+^, Na^+^, Mn^2+^),^29^ and the heterogeneous group of mechanisms behind the import of nuclear-encoded (pre)­proteins.^28,31^ In medicinal chemistry, the latter two functions, driven by ΔΨm + ΔpH, are harnessed as tools for the uptake of lipophilic cations and the import of mitochondria-targeting peptides,^31^ respectively. In early research, it has been established that lipophilic cations penetrate easily through mitochondrial membranes without additional carriers, despite their permanent charge.^21,32^ When linked to functional cargo (therapeutic molecules or reporter dyes) or nanocarriers, with the intent to deliver them to mitochondria, the cationic part of the molecule is referred to as the MTM.^33^ The type of cargo that has been successfully delivered to mitochondria ranges from antioxidants,^34,35^ modulators of mitochondrial therapeutic targets,^20,36,37^ fluorescent dyes,^38^ and even organometallic transfer hydrogenation catalysts.^39,40^

The negative membrane potentials in the plasma membrane (PM) and the inner mitochondrial membrane (IMM) are typically said to be below −30 and −120 mV, respectively (Figure 1).^22,33,41,42^ In rare cases where specifically investigated, the ΔΨm differs greatly depending on whether isolated or live-cell mitochondria are examined.^29^ Physiologically, transient depolarization waves occur at the organelle level as a consequence of spontaneous mitochondrial permeability transition pore (PTP) opening.^43^ In mammalian cells, a drop in ΔΨm is the main trigger for retrograde signaling.^44^ On the other hand, membrane hyperpolarization below −140 mV strongly correlates with increased ROS production,^45^ which itself plays a role in cellular stress adaptation, microenvironmental resistance (both harnessed by cancer cells), and is one of the primary mitochondrial functions.^5^ Stating exact/bottom values of plasma and mitochondrial membrane potentials is therefore not only inexact but also counterproductive. It has been shown that dielectric breakdown of many lipid bilayers can occur at 200 mV of applied voltage; therefore, a ΔΨm beyond −200 mV is improbable.^46^ The formation and lipid composition of the IMM is generally well studied.^47^ It is known that the inner membrane lipids interact with electron transport chain (ETC) proteins and support the formation of cristae. Generation of high ΔΨm also depends on the IMM lipid composition, because mutations in the phosphatidylcholine synthesis pathway resulted in decreased ΔΨm.^48^

**Figure 1:**
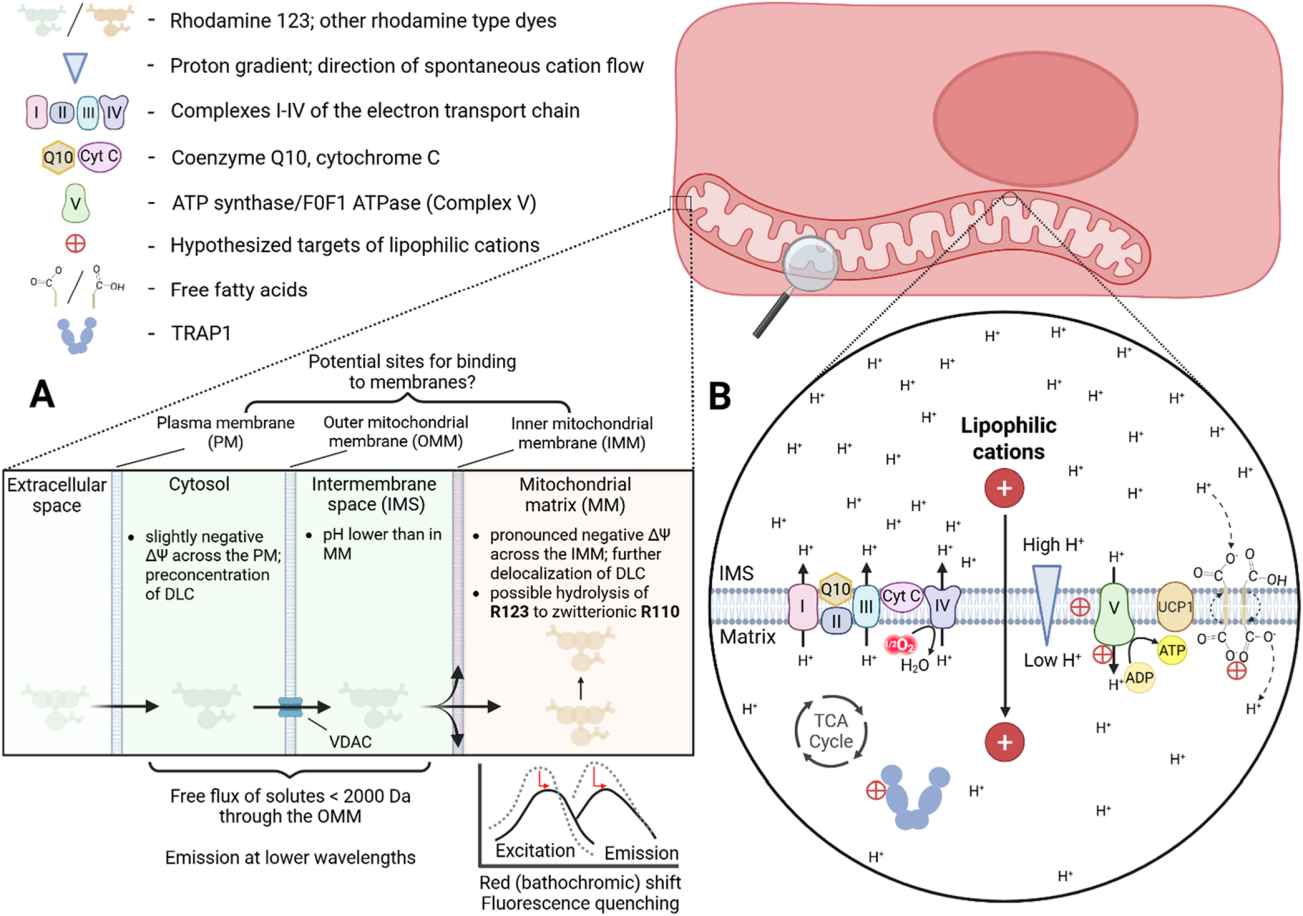
Transmembrane potentials of PM and IMM are harnessed as a free energy source for the import of lipophilic cations. (A) Fluorimetric measurement of ΔΨm takes advantage of the potential-dependent lipophilic cation import, shown here with the example of Rhodamine 123 (R123). PM and IMM drive cation import due to the transmembrane potential (negative inside), and the outer mitochondrial membrane (OMM) allows for free flux of small molecules <2000 Da through the VDAC channel. It is rarely defined whether, and in what quantity, lipophilic cations bind to membranes (PM, OMM, IMM), although mitochondria-targeting antioxidants are said to accumulate and act inside the IMM.^24^ The classical model of passing through cellular compartments to finally reach the MM has been set in early lipophilic cation research,^49^ Several articles touch on the topic of membrane accumulation.^50,51^ Therefore, the membranes are identified as potential sites for cation binding. Rhodamine-cored dyes exhibit a bathochromic shift, as well as fluorescence quenching upon mitochondrial matrix accumulation.^51^ (B) An electrochemical and proton gradient (ΔΨm + ΔpH) is generated by complexes I–IV by expelling protons from the mitochondrial matrix into the intermembrane space (IMS). Cations spontaneously flow into the mitochondrial matrix, resulting in ATP generation (Complex V), heat generation [uncoupling protein-1 (UCP-1), mechanism likely involving long-chain fatty acids],^52^ or lipophilic cation accumulation. Red marks represent some of the hypothesized causes of downstream biological effects observed with lipophilic cations: interacting with the endogenous free fatty acids (FFA), facilitating their protonophorous effect,^21^ swelling/membrane leak (detergent and/or prooxidant effects, present at higher concentrations),^23,24^ F0F1 ATPase inhibition,^27^ TRAP1 ATPase activation/inhibition, dependent on concentration and alkyl chain length. TRAP1 ATPase was not identified in the article as the alkyl-triphenylphosphonium (TPP) binding site.^53^ Selectivity of the distinct MTM structures was not investigated in any of the cited works, leaving the possibility that lipophilic cations share common targets.

The electrochemical potentials across the PM and the IMM are therefore the driving forces for cation uptake, first into the cytosol and then more markedly into the mitochondrial matrix. In the absence of other contributing factors, this process follows the Nernst equation^54^ which states that upon incubation, cations redistribute along the electrochemical gradient in a way that for every 59.2 mV (at standard temperature) or 61.5 mV (at body temperature) of potential, there is a 10-fold increase in concentration of cations on the more negative side of the membrane.^21,30,49,55^ The most prominent deviation from the Nernst equation occurs upon binding of the cationic species to mitochondrial membranes, which results in higher observed accumulation, and in turn, an overestimation of membrane potential if not considered and corrected for.^50,51^ In 1970, a 4-part series of articles titled “Conversion of biomembrane-produced energy into electric form” was published,^49,56–58^ and the authors showed the results of monitoring the transport of organic ions through the IMM to elucidate the mechanism of the generation of ΔΨm. Among the cationic species, methyltriphenylphosphonium was notably used for the experiments. To the best of our knowledge, this is the earliest document of mitochondrial membrane energization leading to the mitochondrial uptake of synthetic lipophilic cations. Another early observation was that lipophilic, membrane-permeable cations accumulate inside energized mitochondria,^49^ and that they can be used as *electric locomotives*—a mitochondrial delivery strategy for noncharged compounds.^59^ This laid the foundation for mitochondria-targeting technology in the following years.

The mitochondrial membrane potential can be determined by measurement of mitochondrial accumulation of mitochondriotropic cations, where potentiometry (i.e., using selective triphenylphosphonium-sensitive electrodes), radiometry (^3^H-labeled tetraphenylphosphonium), and fluorimetry are the main techniques of determining probe concentrations, or their ratios. This is done either in a suspension of isolated mitochondria or in cells in situ.^29^ The mitochondrial membrane potential (ΔΨm) is often stated to be greater in cancer cells compared to normal cells,^34,60^ referencing a group of articles that support this claim, and trace back to the 1980s.^61–64^ Determination of mitochondrial accumulation within these articles is based almost exclusively on Rhodamine 123 (R123) as a cationic fluorimetric probe (For structures of representative fluorescent dyes and reagents used in mitochondrial research, see Figure S1 in the Supporting Information). Compared to its derivatives—tetramethylrhodamine methyl and ethyl ester (TMRM and TMRE)—R123 exhibits, in addition to the expected Nernstian behavior,^51^ significant binding to mitochondrial membranes that contributes to the overall fluorescence intensity independently of ΔΨm. Intracellular/intramitochondrial ester hydrolysis to R110, which retains the measurable fluorescent properties but is likely impermeable due to its zwitterionic nature (Figure 1A—membrane potential measurement using Rhodamine 123), as well as its binding to anions are among the many concerns expressed about R123 as a ΔΨm probe.^55,65^ Its relatively higher retention in cancer cells is not negated, but the critics recommend that conclusions on general cancer cell mitochondrial hyperpolarization be re-evaluated.^29^ Because of its lesser binding to the external mitochondrial surfaces and no observed inhibitory effect on respiration at lower concentrations, TMRM is considered the best choice among the known rhodamine-type dyes.^51^

In more recent experiments, cell staining with JC-1, followed by flow cytometry analysis has revealed that, in some cell lines, varying ΔΨm measurements between subpopulations correlate with phenotypic differences linked to solid tumor expansion, but not with invasion markers.^66,67^ Decreased mitochondrial membrane potential (determined by JC-1 assay in HepG2 and HeLa cells) was found to correlate with radioresistant phenotype.^68^ ^18^F positron emission tomography was used to profile mitochondrial membrane potential heterogeneity within subtypes of lung tumors in vivo.^69^ The increased ΔΨm^70^ is often considered an advantage in anticancer drug development, one of the promising fields for the mitochondria-targeting technology application.^71^ The identified differences in mitochondrial membrane potential of phenotypic tumor subtypes point to a broader picture.^66,67,69^ The techniques at our disposal have improved over the past 40 years, and a novel methodology could greatly improve the state of the art in this aspect.

## Intrinsic Bioactivity of Mitochondria-Targeting Moieties

Triphenylphosphonium (TPP) is a lipophilic cation with a long history of widespread use in mitochondriotropic conjugates, and to this day, it remains the gold standard MTM.^33,72^ The ability of TPP conjugates to accumulate in mitochondria has been proven repeatedly, and tethering functional cargo to TPP has become the default choice to study whether selective delivery of cargo into the mitochondria affects the compound’s biological activity.^33^ The use of the term *mitochondria-targeting moiety* evokes the image of a clean mitochondrial delivery process devoid of any secondary effects. Most evidence point to the fact that all MTMs (including the especially potent TPP), when bound to the otherwise inert lipophilic cargo (such as a short alkyl chain), exhibit a similar bioactivity pattern—including membrane depolarization, induction of apoptosis, and disruption of respiration dynamics, metabolism, and membrane integrity—at high enough (typically low micromolar) concentrations.^21,26,41,73^ The following two examples are especially pertinent: in the first one, a high-throughput MTT-based screening was used to identify TPP attached to prop-1-ene as a potent cytotoxic agent against cancer cell lines.^74^ In the second one, attaching a TPP to an otherwise noncytotoxic functional cargo (nucleobases) resulted in a surge in apoptosis induction and cytotoxicity (an IC_50_ drop from higher values into the low micromolar range).^75^ Although the observed trend of selective toxicity toward cancer versus healthy cell lines cannot be denied, these results still point to the possible contribution of TPP to the nonspecific cytotoxicity when used as an MTM.

When hypothesizing about the concrete mechanisms underlying the biological effects of MTMs,^21,22,41^ a compelling heuristic links lipophilicity and charge distribution to its permeation kinetics, and consequently to its greater concentration in mitochondria. The compound’s structure does influence its ability to distribute into and cross membranes and thus determines the rate and extent of its mitochondrial import. It is therefore true that the permeability and uptake kinetics shape the on-site concentration of the compound and its potency. However, while this framework accounts for the kinetic dimension (how much and how quickly the compound reaches the MM), it fails to capture the qualitative dynamic dimension of the effect. This component and the implied mechanisms all point to the same direction but appear to vary across different MTMs, depending on the experiment. The hypothesized mitochondrial targets highlighted in this chapter are shown in Figure 1B.

At 20 μM, the cetyltrimethylammonium (C_16_TMA) cation has been shown to induce proton leak in mitochondria (isolated from Albino Wistar rat liver tissue) not by acting as an uncoupler, but rather as a detergent which enhanced membrane permeability to all solutes, including protons.^23^ The swelling effect has been reported for MitoQ, and several TPP-plastoquinonyl derivatives (referred to as SkQ, or Skulachev ions), at concentrations exceeding 40 μM, which was attributed to the detergent-like, and prooxidant action.^24^ Molecular dynamics simulations were used to rationalize that despite having similar measured lipophilicities, the C_12_TPP cation has a lower membrane affinity than C_16_TMA.^21^ The difference was attributed to a shorter hydrocarbon chain length, which is further supported by even lower membrane affinities of tetraphenylphosphonium and tetraphenylborate ions, which lack hydrophobic tails altogether. C_12_TPP shows a significantly higher rate constant than C_16_TMA for the membrane transfer, associated with a lower energy barrier of C_12_TPP, presumably due to both charge screening and delocalization. This was also used to explain different oxygen consumption profiles of C_12_TPP and C_16_TMA in State 4 respiration, determined amperometrically (in *Yarrowia lipolytica* isolated mitochondria, concentrations 0–18 μM).^21^ Several proposed mechanisms may be responsible for the difference in oxygen consumption, including F0F1 ATPase inhibition (an early example shown on Rhodamine 123)^27^ and TRAP1 ATPase activation/inhibition as a function of chain length and concentration of TPP bound to a hydrocarbon chain.^53^ Mitochondria-enriched rat skeletal muscle homogenate was incubated with 0.1–1 mM alkyl-triphenylphosphonium cations, demonstrating dose- and chain length-dependent inhibition of complexes I–IV.^26^ The reasoning behind this uncommonly high concentration range was to simulate concentrations in energized mitochondria, a study typically not performed this way, because of concerns about solubility and detergent action of longer-chained TPP derivatives. Some dose–response was nevertheless evident in this study, even though one might expect a plateau at these high concentrations due to physical (solubility) restraints. Another feasible mechanism of altered oxygen consumption rate (OCR) is the facilitation of fatty acids’ protonophorous activity, as shown in an experiment where subuncoupling (0.5 and 2.5 μM) concentrations of C_12_TPP promoted palmitate-induced uncoupling activity with a concentration-dependent EC_50_ shift from 10 μM to 6.0 μM and 2.4 μM, respectively.^21^ Studies show that MitoSOX, a commonly used mitochondria-targeted ROS probe, causes uncoupling and mitochondrial respiration inhibition due to its TPP moiety, and recommend restricting its concentration to 0.2 μM.^76^

Despite the above-discussed possible intrinsic bioactivity of TPP on the reductionist level, TPP-containing conjugates MitoQ,^18^ SkQ1,^19^ and gamitrinib^20^ have been evaluated in humans. This is an important proof of concept for MTM-based therapy.

## Experimental Validation of the MTM–Linker–Cargo Conjugates

Mitochondriotropic conjugates have great potential in medicinal chemistry because of their modular construction and synthetic availability. The moieties that constitute the conjugates (i.e., MTM, linker, and cargo) are to a large extent interchangeable, and the choice of the optimal MTM and linker for a particular cargo depends on the molecular structure and therapeutic indication of the latter. For example, a highly lipophilic cargo might benefit from being part of a conjugate with a less lipophilic MTM and linker to maintain the whole conjugate within an acceptable physicochemical and pharmacokinetic property range. Beyond changing the linker length in alkyl linkers,^42,77–79^ linker chemistry and its effect on mitochondrial accumulation has rarely been studied.^80–82^ Given the recent advancements in “linkerology”, mainly in the field of targeted protein degradation, such as proteolysis-targeting chimeras (PROTACs),^83,84^ and antibody-drug conjugates (ADCs),^85^ optimization of linkers in mitochondria-targeting conjugates to fine-tune physicochemical properties and/or mitochondrial accumulation should also be considered in the future. With respect to the lipophilicity of the permanent cations, the predictive models appear to deviate significantly from the experimentally determined partition coefficients (vide infra). Regarding the intended bioactivity, cyto- and neuroprotective conjugates must not induce cell death at any given therapeutic dose and should therefore include MTM causing the least possible uncoupling and cell death, whereas the anticancer conjugates might synergistically benefit from an MTM with more pronounced intrinsic cytotoxicity.^41^

### The Mitochondrial Accumulation

The mitochondrial accumulation of MTM–cargo conjugates is a primary measure of their efficacy in targeting mitochondria and is studied to prove that the conjugates selectively reach their mitochondrial targets in sufficient concentration, which accompanies and relates to the results of phenotypic, cell-based assays.^86^ To compare the extent of accumulation within a series, the triphenylphosphonium MTM is often used as a benchmark.^22,41,72^ The methods for quantifying mitochondrial accumulation fall into two distinct categories: accumulation of MTM–(imaging cargo) conjugates and accumulation of MTM–(functional cargo) conjugates. Methods for determining mitochondrial accumulation of fluorescent dyes are somewhat related to the methods of determining ΔΨm, given that the same mechanism of uptake applies to both and that the signal can be read out from intact cells. For novel MTMs, colocalization metrics—such as the Pearson correlation coefficient and Manders’ overlap coefficients—along with relative fluorescence intensity, can be assessed through coincubation with a validated mitochondrial dye (e.g., a dye from the MitoTracker series, see Figure S1, Supporting Information) at a fixed concentration and incubation time. Monitoring both colocalization and fluorescence intensity under these controlled conditions allows for the generation of comparable measurements across different MTM conjugates.^72^

The challenge with the quantitative assessment of mitochondrial concentration of functional (e.g., therapeutic) cargo–MTM conjugate is that only a small subset of mitochondria-targeted functional conjugates provides an electromagnetic signal suitable for spectroscopic measurement (e.g., visible light absorption, fluorescence, or γ ray emission), and out of the fluorescent compounds only a subset emits wavelengths that do not interfere with cellular autofluorescence. An exception to this rule are theranostic agents^87^ and photodynamic therapeutic agents.^88^ Therefore, for a vast majority of nonfluorescent MTM conjugates, any in situ measurements of mitochondrial localization within the intact cells cannot be performed. MTM conjugates with the heat shock protein 90 (Hsp90) inhibitor 17-AAG, including several cyclic guanidinium and a triphenylphosphonium-type lipophilic cations—collectively named Gamitrinibs—are clinically evaluated mitochondria-targeted anticancer agents.^20^ Their accumulation in the mitochondrial extract isolated from HeLa cells was demonstrated by gradient density centrifugation, which showed increased absorbance of the Gamitrinib-loaded mitochondria in comparison with the parent 17-AAG (nonconjugated to MTM), which remained mostly in the upper nonmitochondrial fractions.^89^ The gradient density centrifugation approach does not allow for a comparison of relative concentrations in mitochondria, cytosol, and other cellular compartments. This type of study is possible only if the intact cells are first incubated with the compound of interest and then subcellularly fractionated. For Gamitrinib–TPP, a 106:1 ratio of the mitochondrial and cytosolic concentration was shown by UHPLC/MS analysis upon fractionation of PC3 cells.^86,90^

According to the Nernst equation, the mitochondrial concentration of cations is estimated to be 100–1000 times greater than the extracellular levels. However, these values are occasionally cited without full context, which can lead to misunderstandings.^75,91,92^ The equation does not account for contributions other than ΔΨm, which depend on the structure of the MTM and the conjugate as a whole.^78^ The experimentally observed large variability in mitochondrial uptake of various cationic probes implies that not just any kind of cationic species can be considered an efficient MTM, with pure Nernstian behavior.^21^ Furthermore, translocation back into the cytosol and extracellular medium once the membrane potential is dissipated due to uncoupling has also been reported.^33^ Another proof that the extent of accumulation is dependent on the MTM-conjugate structure was shown by incubation of the isolated mitochondria with the tested conjugates, followed by measuring the concentration decrease in the supernatant:^22^ In their experimental setup, the conditions of no mitochondrial uptake were first generated (mitochondrial membrane potential dissipated by Complex I inhibitor rotenone), followed by the membrane potential-induced uptake (generation of membrane potential in the presence of Complex II substrate—10 mM succinate), which was again reversed by membrane potential dissipation (using FCCP as uncoupler).^15^ The advantage of this method is that it allows for a semiquantitative measurement within the MTM series with sufficiently high throughput and bypasses the need for MTM–(fluorescent dye) conjugates, because the aqueous supernatant medium is suitable for standard HPLC analysis with DAD detection. The method, however, does not account for the presence of the plasma membrane, which has its own membrane potential and lipid composition (see Figure 1A), and is therefore less physiologically relevant than experiments in intact cells.

Exact determination of mitochondrial concentration of any mitochondriotropic conjugate is utopian because some essential parameters are still difficult to determine in practice: the exact mitochondrial volume and count in individual experiments, cytoplasmic volume, and the extent of adsorption to and accumulation in membranes, all of which depend on the cell line and conditions outside the cell. For the purpose of (semi)­quantitative evaluation of conjugates with various MTMs, exact determination of concentration is not essential as long as the MTMs are benchmarked against the TPP conjugates with the same linker and cargo in the same concentration, within the same cell line.

### The Bioactivity of MTM Conjugates

The bioactivity of MTM conjugates with functional (bioactive), inert, or fluorescent cargo can be evaluated by cell-based (phenotypic) assays. However, if a novel mitochondriotropic is developed, despite giving a biological signal, the on-target activity or functionality of the parent functional cargo may or may not have been retained upon attaching the MTM. The activity should therefore be re-evaluated for every novel MTM attached to functional cargo, and especially for novel classes of functional cargos.^39,89,93–95^ Figure 2 provides a graphical overview of the methods historically used in MTM research and those still relevant today to quantify the emergent bioactivity of MTM–cargo conjugates (vide infra), including Seahorse extracellular flux (XF) assay,^96^ JC-1, JC-10,^97^ or TMRM assay,^89^ MitoSOX assay,^98,99^ PTP opening assays,^60,89,100^ annexin V assay,^101^ propidium iodide assay,^102,103^ membrane integrity assays, MTT assay, and resazurin assay. The most commonly used methods for assessing MTM-induced effects have been grouped into four categories: mitochondrial function assays (Figure 2A), cell death assays (Figure 2B), membrane integrity assays (Figure 2C), and metabolic activity assays (Figure 2D). The latter two types of assays are often referred to as “viability studies,” although the three events (loss of membrane integrity, metabolic activity, and viability) do not necessarily coincide in cells.^104^

**Figure 2:**
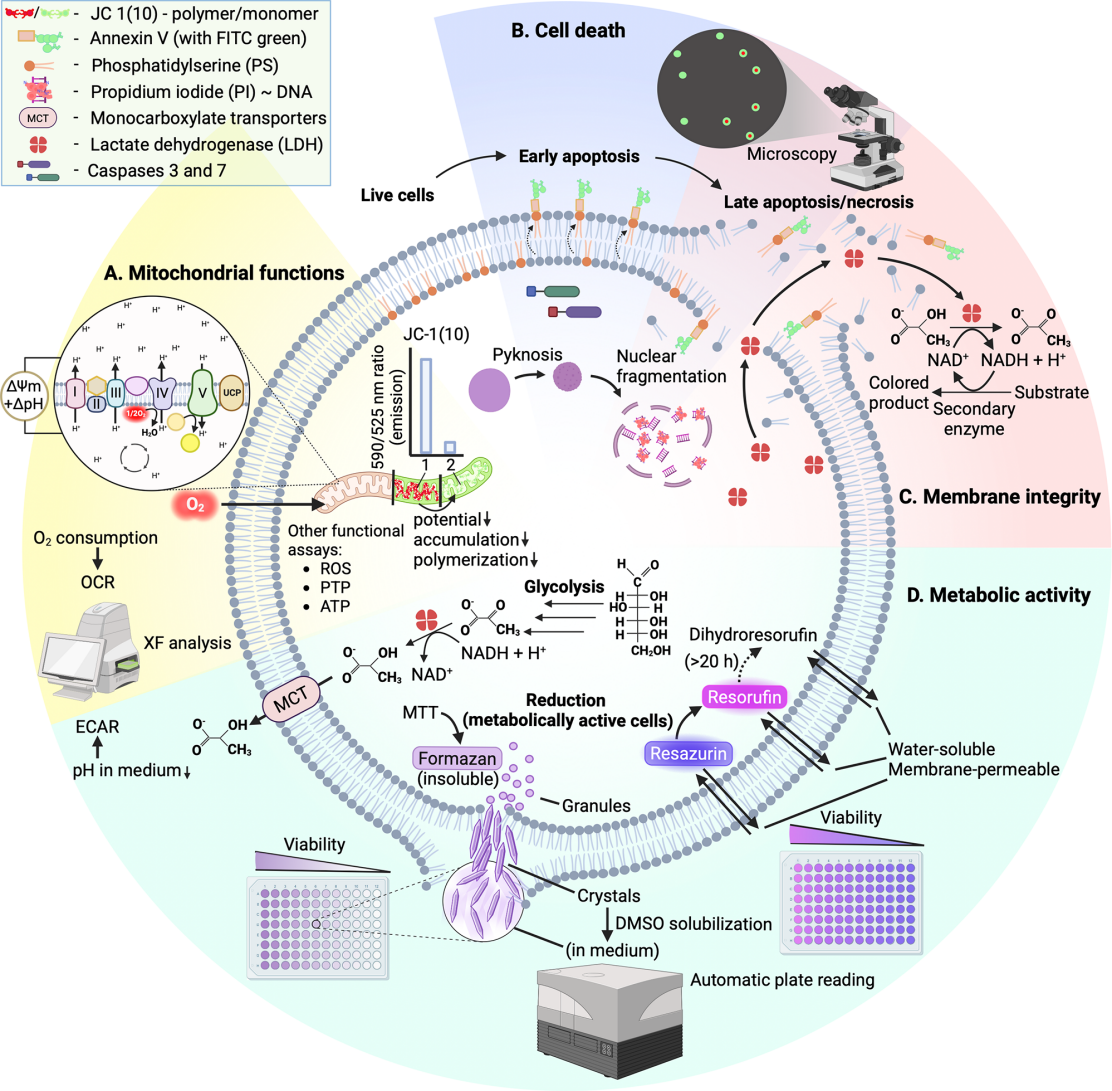
Overview (chemist’s survival guide) of the cell-based assays to assess the biological effects of conjugates with MTMs.^105^ The assays are categorized into four main categories (A–D), based on the type of cell properties being assessed. (A) Mitochondrial functions. The Seahorse XF can analyze the oxygen consumption rate (OCR) using the “mito stress” test. The measures obtained (altered by treatment-induced effects) are basal respiration, ATP production, proton leak, and maximal respiration.^96^ The JC-1 (or JC-10) assay gives information on mitochondrial membrane potential, based on the ratio of 590 nm (higher aggregation of dye) to 525 nm (lower aggregation of dye) emission. The two have some overlap and complement each other, for example, when assaying depolarization (JC-1 assay) that is caused by uncoupling (XF analysis). Membrane potential changes can be measured using other dyes, such as TMRM.^89^ ROS generation can be measured fluorimetrically using the MitoSOX dye, a common mitochondria-targeted ROS probe selectively oxidized by superoxide.^98,99^ ATP production rate can be measured in real time using OCR and extracellular acidification rate (ECAR) data obtained from the Seahorse XF Analyzer, comparing ATP produced from oxidative phosphorylation and glycolysis.^106^ Effect on permeability transition pore (PTP) opening is measured either in terms of mitochondrial depolarization^89^ or swelling^60^ caused by PTP opening (reversed by cyclosporin A, a selective PTP inhibitor), or inhibition of PTP opening (cyclosporin A used as a positive control in a Ca^2+^ tolerance assay).^100^ (B) Cell death. Caspase-3 and -7 are executioner caspases that can be used in fluorescent microscopy to continuously monitor apoptosis by activating reagents such as Incucyte Caspase-3/7, and releasing a fluorescent DNA-intercalating dye.^107^ Annexin V binds to phosphatidylserine exposed in the outer phospholipid layer of the cell membrane during early apoptosis. It is often duplexed with propidium iodide (membrane integrity assay) which binds to nucleic acids upon nuclear fragmentation, and thus helps distinguish between early and late apoptosis.^103,103^ (C) Membrane integrity. Another technique of determining disruption in membrane integrity are assays that use cytosolic enzymes, adding external substrates/cofactors which can be more selectively quantified by a secondary reaction (e.g., adding cofactor NAD^+^, resazurin substrate and a secondary enzyme that gives a colored product using formed NADH as a cofactor). (D) Metabolic activity. “Viability assay” is the common name for MTT (or MTS) and resazurin assays which quantify the activity of cell reductases. Glucose is metabolized to pyruvate, which is converted to lactate and H^+^, and the Seahorse XF can be used to analyze ECAR, a proxy for glycolysis activity (measure of Warburg effect features).^96^

## Notable Campaigns for the Development of Novel MTMs beyond TPP

Efforts toward the development of improved MTMs mainly follow three goals: (i) improving the mitochondria-targeting ability of the cationic MTM, (ii) decreasing unwanted intrinsic bioactivity of MTM, or (iii) improving physicochemical properties of the conjugate. Charge delocalization has frequently been discussed as a key parameter influencing the membrane permeability of cationic MTMs,^37,60,72,108^ although “localized” permanent cations such as trialkylphosphonium have also been shown experimentally to accumulate efficiently in mitochondria.^94,109,110^ Experimental^37^ and computational approaches^72^ were used to explain the effect of charge distribution on membrane permeability and mitochondrial localization. Systematic investigations correlating (experimentally determined or modeled) charge distribution on the cationic MTMs with cellular uptake, mitochondrial accumulation, and intrinsic bioactivity would further advance our understanding in this area.

According to the property forecast index (PFI = log*D*_7.4_ + #Ar),^111^ a high number of aromatic rings in the bioactive molecule is detrimental to its drug-like properties. From this viewpoint, switching from TPP to, for example, the tricyclohexylphosphonium cation would be beneficial. The mitochondrial accumulation of trialkylphosphonium cations was systematically compared to the triphenylphosphonium cation.^110^ The butyl-tricyclohexylphosphonium bromide **1** (Figure 3) displayed a similar membrane potential-dependent accumulation in isolated rat liver mitochondria, then the TPP benchmark, determined by a TPP-selective electrode, while exhibiting slightly higher experimentally determined octanol–water partition coefficient. Moreover, the uptake of tricyclohexylphophonium cation conjugated with fluorescein (**2**) to isolated rat liver mitochondria was similar to the TPP–fluorescein conjugate, determined by fluorescence correlation spectroscopy.^110^ The mitochondria-targeting ability of the tricyclohexylphosphonium cation was also demonstrated for its conjugate with BODIPY-type fluorescent dye **3** by using laser-scanning confocal microscopy. In particular, colocalization with MitoTracker Deep Red in intact HeLa cells was demonstrated.^109^ Tricyclohexylphosphonium conjugate with TRAP1 inhibitor **4**, compared to its TPP analog retained the on-target activity, and has in vitro activity against cancer (HeLa) and normal (human foreskin fibroblast) cell line in a comparable range in the MTT assay.^94^ Tricyclohexylphosphonium cation was also used as MTM, conjugated to an oleanolic acid derivative,^112^ chlorambucil,^113^ and to a luminol derivative.^114^ Based on the data presented above, the tricyclohexylphosphonium cation appears to possess mitochondria-targeting ability comparable to that of TPP. However, systematic studies of ADME-related physicochemical properties and the intrinsic bioactivity of saturated versus aromatic phosphonium cations are still required to assess their potential for improved drug-likeness.

**Figure 3:**
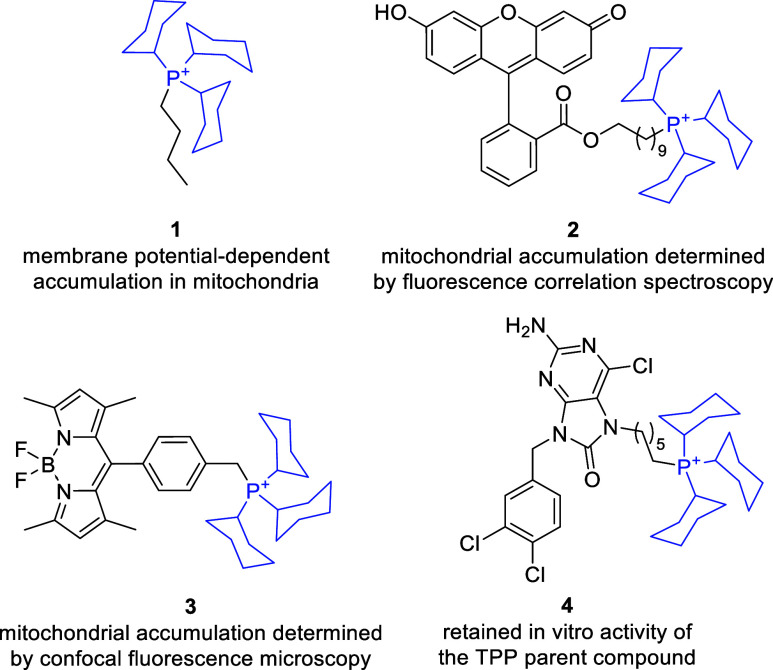
Tricyclohexylphosphonium cations as mitochondria-targeting moieties.

Adding substituents to the aromatic rings of the TPP moiety affects the mitochondria-targeting ability and intrinsic bioactivity of the resulting triarylphosphonium cations by influencing the charge distribution and lipophilicity. It should be emphasized that the predicted lipophilicity (clog*P*) for triarylphosphonium cation conjugates, and for permanent cations in general, can be considerably higher compared with the experimentally determined octanol/buffer partition coefficients. For example, the fluorescein conjugate **5** has an experimental log*D*_7.4_ = 2.37 ^42^ (determined by shake-flask method in 1-octanol/phosphate buffer), whereas its predicted clog*P* values are 14.4 (calculated in ChemDraw Professional 22) and 8.0 (consensus log*P*, calculated in SwissADME).^115^ The implicit solvation model-based log*P* prediction method (iLOGP)^116^ seems to give better correlation with the experimentally determined log*D* values for triarylphosphonium cations; the predicted iLOGP value for **5** is 2.8.

Generally, it has been shown that adding alkyl substituents to the aromatic rings of TPP results in increased lipophilicity,^42,117–120^ and that increased lipophilicity of the whole conjugate correlates with increased cellular uptake and increased mitochondrial localization,^42^ as well as with increased cytotoxicity, see for example fluorescent conjugates **5** and **6** (Figure 4). While MTMs with emergent bioactivity may find application in anticancer MTM–drug conjugates, more inert MTMs are generally desired for mitochondrial delivery of functional cargo. A systematic study of *P*-alkylated triphenylphosphonium cations with electron-donating or electron-withdrawing *para* substituents on the phenyls revealed that tris­(*p*-CF_3_-phenyl)­phosphonium might be a less intrinsically bioactive MTM compared to the bare TPP.^41^ Indeed, the electron-poor *P*-decyl-tris­(*p*-CF_3_-phenyl)­phosphonium bromide **7**, compared to the TPP conjugate, displayed lower dissipation of ΔΨm (determined by JC-1 assay), lower cytotoxicity (determined by MTT assay in C2C12 mouse myoblast cells), and lower uncoupling of oxidative phosphorylation (determined by Seahorse XF mito stress test assay in C2C12 cells). On the other hand, the electron-rich *P*-decyl-tris­(*p*-OMe-phenyl)­phosphonium bromide **8** was found to be more cytotoxic, a stronger uncoupler, and caused more ΔΨm dissipation than the TPP benchmark. **7** was shown by a TPP ion-selective electrode to accumulate in the isolated mouse hindlimb mitochondria in a ΔΨm-dependent manner. Moreover, a conjugate with fluorescent dye TAMRA **9** was prepared in order to evaluate the ability of tris­(*p*-CF_3_-phenyl)­phosphonium cation to deliver net-neutral cargo into mitochondria; colocalization with MitoTracker Green was used to determine mitochondrial delivery of **9** by fluorescence microscopy.^41^ Instability of **7** in alkaline aqueous DMSO solution was described; however, the compound was found stable in most other tested biologically relevant conditions.^121^ This is an important observation, because the first choice for most in vitro experiments is diluting the compound from DMSO stock into buffer at pH > 7, conditions irrelevant for potential future clinical use. A conjugate of tris­(*p*-CF_3_-phenyl)­phosphonium cation and metformin **10** was prepared and was found to exhibit a favorable in vivo safety profile compared to the TPP conjugate. In the same study, ^19^F NMR was used to detect **10** in vitro and ex vivo.^122^

**Figure 4:**
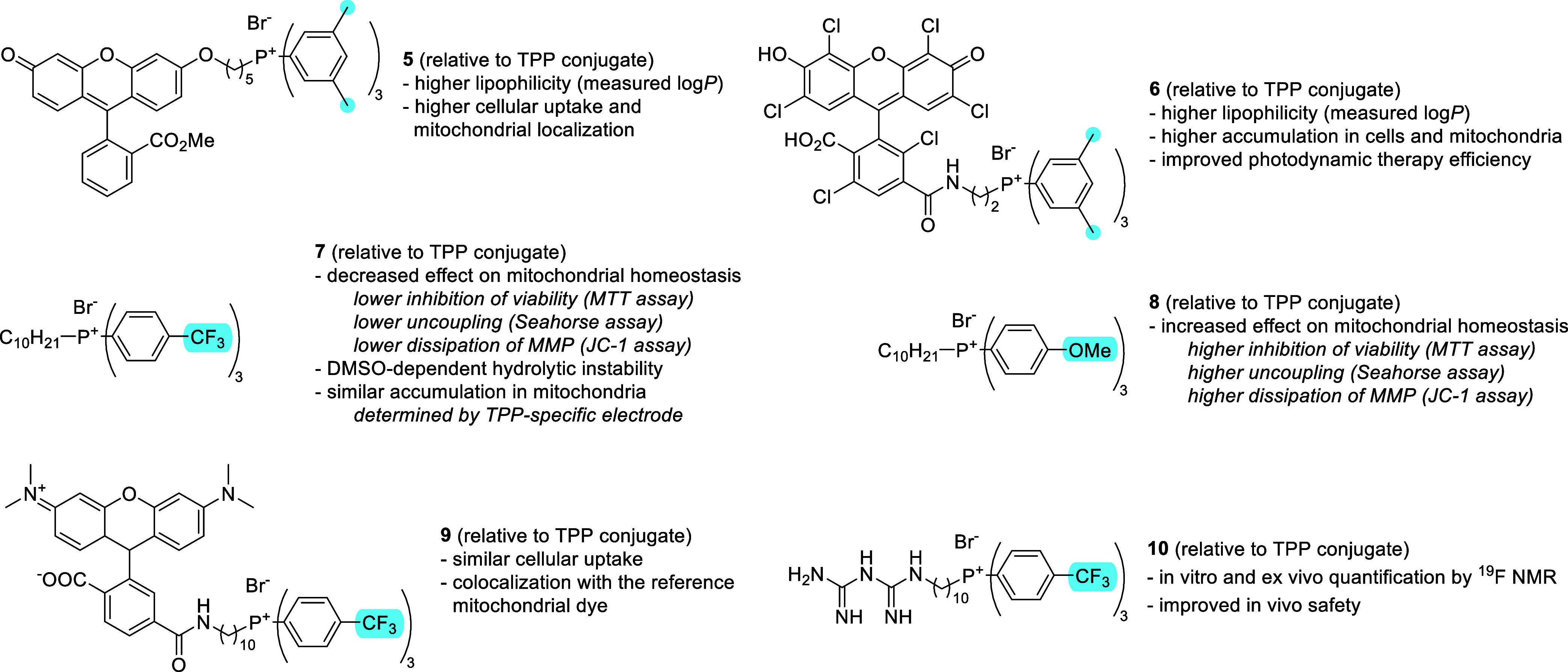
Aryl-substituted triphenylphosphonium cations as mitochondria-targeting moieties.

*P*-Amino-triphenylphosphonium cations were developed as novel MTMs that showed superior accumulation in mitochondria compared to TPP (Figure 5).^72^ Their mitochondria-targeting ability was quantified by confocal microscopy of the conjugates with fluorescein methyl ester using MitoTracker Deep Red as a reference mitochondrial dye. The conjugate with new aminophosphonium cation **11** displayed better cell accumulation (fluorescence intensity ratio of fluorescein and MitoTracker Deep Red channel) and better mitochondrial localization (Pearson and Manders coefficients of fluorescein and MitoTracker channels) than the benchmark TPP conjugate **12**. The authors proposed the maximum of the molecular electrostatic potential surface of the cations, V_s,max_, as a molecular descriptor correlating with mitochondrial accumulation.^72^ It may simply be that the higher observed accumulation of the fluorescent probe **11** compared to **12** is a function of lipophilicity: if we compare the microscopy-derived accumulation parameters of the *P*-amino-triphenylphosphonium **11** with its iso-lipophilic triarylphosphonium analog **5**,^42^ their cell and mitochondrial accumulation is in the same range. Conjugates of the new aminophosphonium MTMs with short alkyl chains (inactive cargo) were also evaluated for their cytotoxicity (HeLa, resazurin assay) and lipophilicity (experimentally determined octanol/water partition coefficient, log*P*), see Figure 5. Cytotoxicity seems to correlate with lipophilicity; the more lipophilic aminophosphonium **13** and triarylphosphonium **14** have submicromolar IC_50_ values, whereas the cytotoxicity of the TPP analog **15** is an order of magnitude lower. This points to a rather high intrinsic cytotoxicity of aminophosphonium- and alkylated triphenylphosphonium-based MTMs used in compounds **11**–**14**. On the other hand, if we compare compounds **16**–**18** which all have the same log*P* value, the cations present in **16** and **17** might have potential as MTMs with decreased intrinsic bioactivity relative to TPP, but first, their ability to deliver fluorescent and/or bioactive cargo to mitochondria would have to be validated.

**Figure 5:**
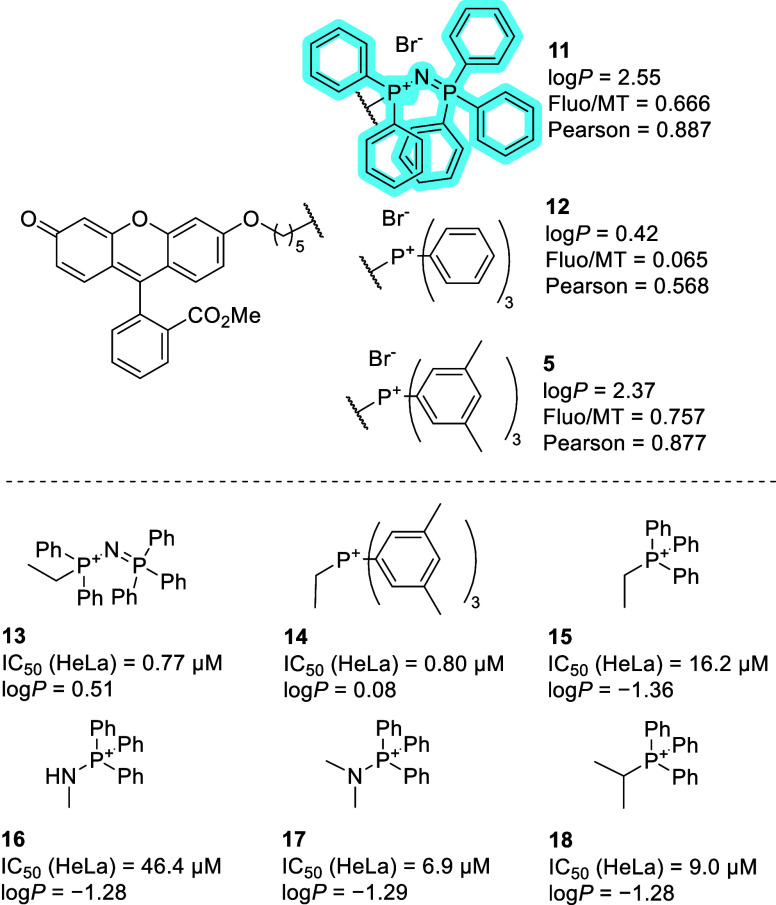
*P*-Amino-triphenylphosphonium cations as MTM,^60^ and their comparison to *P*-alkyl-triarylphosphonium cations.^33^ log*P* values were determined for *n*-octanol/water by the shake-flask method. IC_50_ values on HeLa cells were determined after 72 h by the resazurin assay. Fluo/MT represents the fluorescence intensity ratio of fluorescein and MitoTracker Deep Red channel.

The TPP-pegylated BODIPY dyes **19** and **20** were compared to bare triphenylphosphonium conjugate **21** (Figure 6) and to the tris­(cyclohexyl)­phosphonium conjugate (compound **3**, Figure 3).^109^ Pegylation was not detrimental to the mitochondria-targeting ability within the cell: **19** and **20** exhibited a high degree of colocalization with the reference mitochondrial dye MitoTracker Deep Red in HeLa cells. However, the cellular uptake of the more polar **19** and **20** was much lower than that of **21** and **3**. As a qualitative measure, to achieve a similar fluorescence intensity, MitoTracker Deep Red was used at 0.1 μM, **21** and **3** at 0.5 μM, **19** at 5 μM, and **20** at 25 μM. The decreased cellular uptake correlated with decreased cytotoxicity in HeLa cells.^109^ The pegylated compounds **19** and **20** can therefore be considered biocompatible mitochondria-targeted fluorescent probes. The aryl-pegylated triphenylphosphonium cations may find application in mitochondrial delivery of very lipophilic cargo (including and beyond fluorescent dyes), where the balanced lipophilicity of the whole conjugate would not preclude cellular uptake.

**Figure 6:**
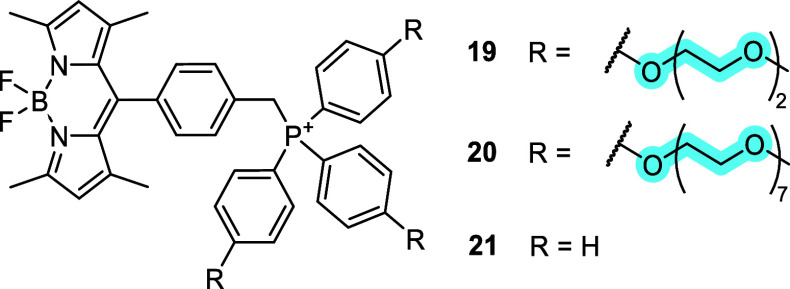
Conjugates of BODIPY-based fluorescent dyes with pegylated triphenylphosphonium cations.

A modular synthetic strategy with a late-stage introduction of MTM was used to prepare a series of caffeic acid conjugates with TPP (**22**), and nitrogen-based heterocycles (**23**–**25**), see Figure 7.^22^ The conjugates were assayed for cytotoxicity in differentiated SH-SY5Y and HepG2 cells (as changes in metabolic activity, lysosomal activity, and cell mass), uptake to isolated mouse liver mitochondria, influence on cell respiration and proton leak (Seahorse XF assay), ΔΨm dissipation (JC-1 assay), and cell death induction (annexin V and propidium iodide assays). The nitrogen-based MTMs (**23–25**) were found to be significantly less cytotoxic compared to the TPP conjugate **22**, and also exhibited less bioactivity in Seahorse XF and JC-1 assays.^22^ Neuroprotective effect due to the antioxidant property of the caffeic acid moiety was evaluated in SH-SY5Y cells. The quinolinium conjugate **23** was identified as the most promising nitrogen-based MTM, because at 50 μM, it exhibited neuroprotective effect against *tert*-butylhydroperoxide similar to the TPP conjugate **22** at 10 μM. The pyridinium conjugate **24** and imidazolium conjugate **25** were significantly less effective as neuroprotective agents against *tert*-butylhydroperoxide at 50 μM in SH-SY5Y cells.^22^ The overall results indicate that all tested nitrogen-based MTMs (**23**–**25**) exhibit efficient mitochondrial-membrane-potential-dependent accumulation in isolated mitochondria. On the other hand, the cellular uptake of nitrogen-based MTM conjugates was not directly assessed. The lower (desired and undesired) bioactivities of **23**–**25** relative to **22** might therefore be a consequence of their lower cellular uptake. Exploring a range of bioactive cargo conjugates with nitrogen-based MTMs would provide a clearer assessment of their overall potential.

**Figure 7:**
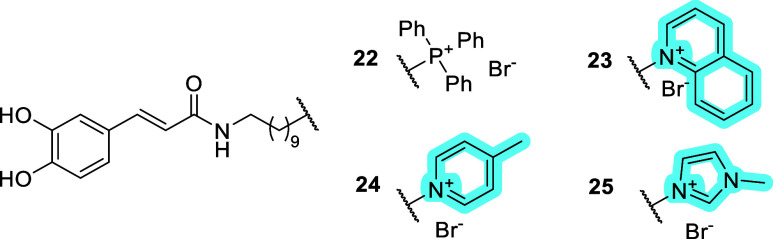
Conjugates of caffeic acid with TPP and nitrogen-based MTMs.

An exemplar development campaign was demonstrated for the mitochondria-targeted cyclosporin A (CsA) conjugates (Figure 8).^37,93,123,124^ CsA is an immunosuppressive drug that binds to cyclophilins. Selective inhibition of the mitochondrial isoform cyclophilin-D (CyP-D) on a cellular level was achieved by attaching TPP as MTM. The linker and MTM were attached to CsA via α-alkylation of cyclosporin’s glycine moiety. In addition to improved selectivity over the cytosolic cyclophilin-A, the mitochondria-targeted cyclophilins exhibited improved cytoprotection in oxygen and/or glucose-deprived hippocampal neurons.^93^ The follow-up compound **26** with an optimized linker exhibited an improved CyP-D affinity.^123^ After the TPP-based proof-of-concept compounds, the authors switched the MTM to quinolinium cation due to TPP’s, in their words, “*non-ideal pharmaceutical properties*”. The quinolinium compound **27** was prepared by olefin metathesis on the cyclosporine A and was suitable for in vivo experiments in murine multiple sclerosis model showing significant neuroprotection with minimized immunosuppressive effects of the parent CsA.^124^ The bioactivity profile of **27** indicates that quinolinium can act as an MTM in vitro and in vivo, although the authors did not show an unambiguous experimental confirmation of mitochondrial localization of **27** or of the quinolinium conjugate with fluorescent dye. The quinolinium MTM was then further optimized in a series of CsA conjugates.^37^ The frontrunner compound **28** has improved pharmacokinetic properties relative to **27**, in particular, higher concentration in brain. Notably, the morpholino-substituted compound **28** shows higher plasma and brain concentrations in mice compared to the unsubstituted **27**, while the brain-to-plasma concentration ratio remains similar for both compounds, at approximately 2%.^37^

**Figure 8:**
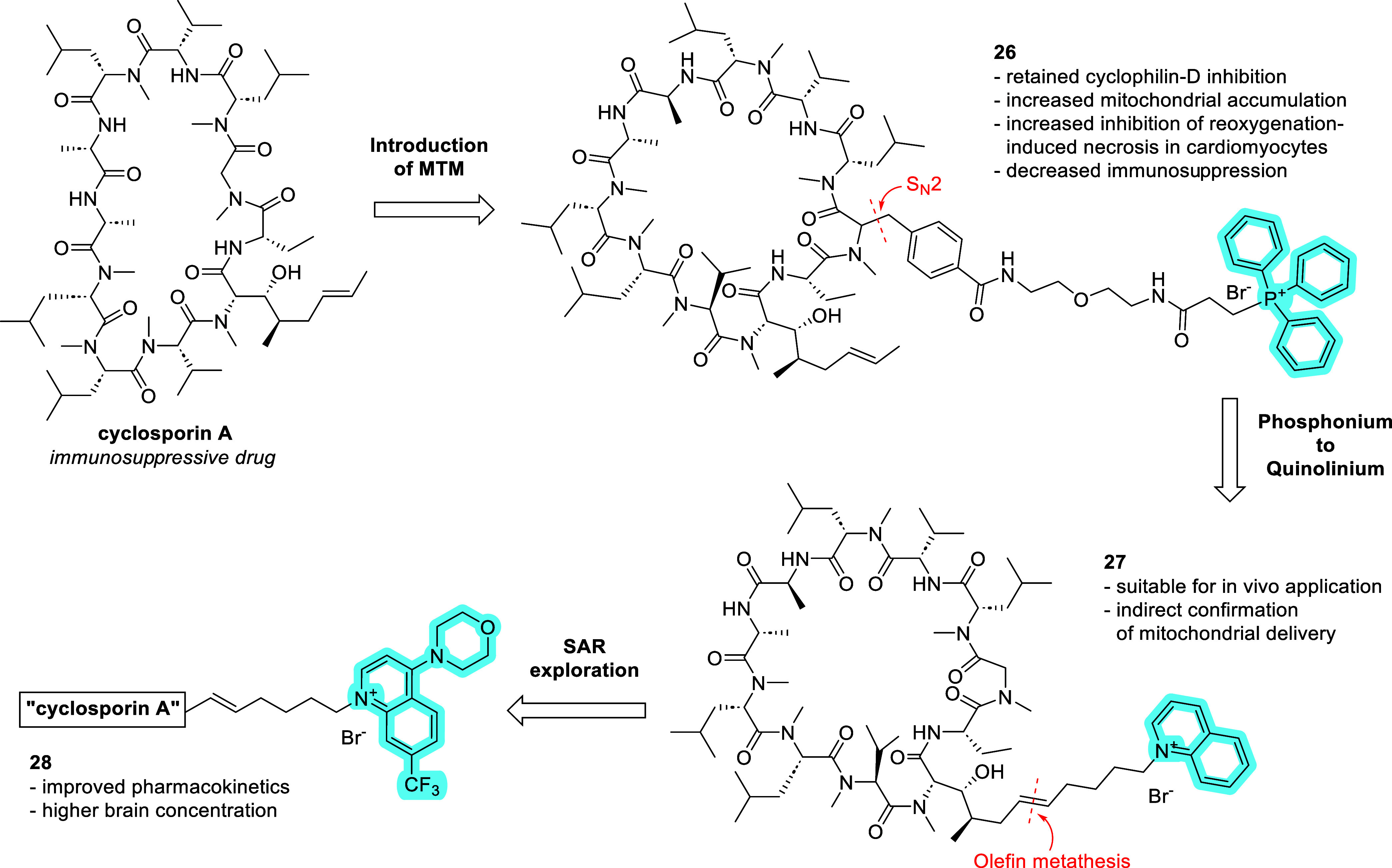
Development of mitochondria-targeting cyclosporin analogs.

## Conclusions and Future Perspectives

Mitochondria are central coordinators of cellular physiology, extending beyond their canonical role in ATP generation to encompass signaling, metabolism, and regulation of cell survival and death. This complexity presents both opportunities and challenges for drug discovery. Clinical setbacks with mitochondrial metabolism inhibitors such as IACS-010759 and CPI-613 highlight the difficulty of achieving efficacy and tolerability, yet the recent FDA approval of the ClpP activator dordaviprone demonstrates that carefully designed strategies can achieve clinical success. Moving forward, translational progress will depend on therapeutic concepts that explicitly account for the multifaceted and context-dependent nature of mitochondrial biology rather than relying on reductionist assumptions.

Within this conceptual framework, an attractive modular strategy for the delivery of bioactive small molecules specifically to mitochondria is conjugation to MTMs via a covalent linker. We emphasize that all MTMs, including and especially the archetypal TPP exert biological effects independent of their cargo, and this intrinsic bioactivity must be considered in drug design. Several notable campaigns for the development of novel MTMs beyond TPP have been published recently, some focusing on improved mitochondrial delivery and others on reduced intrinsic bioactivity or improved physicochemical/pharmacokinetic properties.

Ideally, instead of defaulting to TPP, the developers of future mitochondria-targeting bioactive molecules will have a broader selection of validated MTMs to choose from, supported by experimental evidence of their suitability. To that end, we propose an experimental framework for characterizing structurally novel MTMs, involving three types of conjugates:

(a) **Conjugate with an imaging cargo** to quantitatively assess mitochondrial delivery. For the fluorescent cargo conjugates, the dye should be net neutral, lack pre-existing organelle specificity, and possess adequate photophysical properties to allow use at low concentrations without interference from cellular autofluorescence. The dye conjugates used in the literature examples discussed herein were derived from fluorescein,^42,72,110,124^ rhodamine,^41^ or BODIPY.^109^
(b) **Conjugate with an inert cargo** to evaluate the intrinsic bioactivity of the MTM. Alkyl chains of varied lengths, such as butyl and decyl, seem to be appropriate model otherwise inert cargo.^41^
(c) **Conjugate with a functional cargo** to assess mitochondrial delivery of a pharmacologically active agent and induce biological activity within mitochondria. Particularly informative are conjugates containing modulators of mitochondrial targets, for which organelle localization is critical for their distinct bioactivity, such as cyclophilin inhibitors^93^ or heat shock protein inhibitors.^94^

Because absolute measures of mitochondrial accumulation and MTM-specific bioactivity remain difficult to define, benchmarking new candidates against TPP within the same experimental system is both practical and informative.

We believe that the success of future MTM–linker–cargo therapeutics (**MITACs**, **MIT**ochondria-**TA**rgeting **C**onjugates) will depend on the precise pairing of each cargo with its MTM and linker, guided by the clinical context and physicochemical considerations. Achieving this will require a harmonized experimental approach and a more holistic understanding of MTM behavior. In doing so, the field can move beyond one-size-fits-all solutions toward broader and more tailored applications.

## Supplementary Material



## Notes

Structures of some fluorescent dyes and reagents used in mitochondrial research (Figure S1) (PDF)

## Abbreviations Used

ADC: antibody–drug conjugate
CsA: cyclosporin A
CyP-D: cyclophilin D
ECAR: extracellular acidification rate
ETC: electron transport chain
FFA: free fatty acid
Hsp90: heat shock protein 90
IMM: inner mitochondrial membrane
IMS: intermembrane space
MITAC: mitochondria-targeting conjugate
MTM: mitochondria-targeting moiety
OCR: oxygen consumption rate
OMM: outer mitochondrial membrane
PFI: property forecast index
PM: plasma membrane
PROTAC: proteolysis-targeting chimera
PTP: permeability transition pore
R123: Rhodamine 123
SkQ: Skulachev ion
TMRM: tetramethylrhodamine methyl ester
TMRE: tetramethylrhodamine ethyl ester
TPP: triphenylphosphonium
VDAC: voltage-dependent anion channel
XF: extracellular flux

## References

[1] Yang J., Griffin A., Qiang Z., Ren J. (2022). Organelle-Targeted
Therapies: A Comprehensive Review on System Design for Enabling Precision
Oncology. Signal Transduction Targeted Ther..

[2] Mukherjee S., Bhatti G. K., Chhabra R., Reddy P. H., Bhatti J. S. (2023). Targeting
Mitochondria as a Potential Therapeutic Strategy against Chemoresistance
in Cancer. Biomed. Pharmacother..

[3] Liu Y., Shi Y. (2020). Mitochondria as a Target
in Cancer Treatment. MedComm.

[4] Siekevitz P. (1957). Powerhouse
of the Cell. Sci. Am..

[5] Palma F. R., Gantner B. N., Sakiyama M. J., Kayzuka C., Shukla S., Lacchini R., Cunniff B., Bonini M. G. (2024). ROS Production by
Mitochondria: Function or Dysfunction?. Oncogene.

[6] Wescott A. P., Kao J. P., Lederer W. J., Boyman L. (2018). Regulation of ATP Production
by Mitochondrial Calcium Signals in Heart. Biophys.
J..

[7] Biasutto L., Azzolini M., Szabò I., Zoratti M. (2016). The Mitochondrial Permeability
Transition Pore in AD 2016: An Update. Biochim.
Biophys. Acta, Mol. Cell Res..

[8] Weber-Lotfi F., Koulintchenko M. V., Ibrahim N., Hammann P., Mileshina D. V., Konstantinov Y. M., Dietrich A. (2015). Nucleic Acid Import into Mitochondria:
New Insights into the Translocation Pathways. Biochim. Biophys. Acta, Mol. Cell Res..

[9] Muneretto G., Plazzi F., Passamonti M. (2024). Mitochondrion-to-Nucleus
Communication
Mediated by RNA Export: A Survey of Potential Mechanisms and Players
across Eukaryotes. Biol. Lett..

[10] Stine Z. E., Schug Z. T., Salvino J. M., Dang C. V. (2022). Targeting Cancer
Metabolism in the Era of Precision Oncology. Nat. Rev. Drug Discovery.

[11] Sainero-Alcolado L., Liaño-Pons J., Ruiz-Pérez M.
V., Arsenian-Henriksson M. (2022). Targeting
Mitochondrial Metabolism for Precision Medicine in Cancer. Cell Death Differ..

[12] Murphy M. P., Hartley R. C. (2018). Mitochondria as
a Therapeutic Target for Common Pathologies. Nat. Rev. Drug Discovery.

[13] Yap T. A., Daver N., Mahendra M., Zhang J., Kamiya-Matsuoka C., Meric-Bernstam F., Kantarjian H. M., Ravandi F., Collins M. E., Francesco M. E. D., Dumbrava E. E., Fu S., Gao S., Gay J. P., Gera S., Han J., Hong D. S., Jabbour E. J., Ju Z., Karp D. D., Lodi A., Molina J. R., Baran N., Naing A., Ohanian M., Pant S., Pemmaraju N., Bose P., Piha-Paul S. A., Rodon J., Salguero C., Sasaki K., Singh A. K., Subbiah V., Tsimberidou A. M., Xu Q. A., Yilmaz M., Zhang Q., Li Y., Bristow C. A., Bhattacharjee M. B., Tiziani S., Heffernan T. P., Vellano C. P., Jones P., Heijnen C. J., Kavelaars A., Marszalek J. R., Konopleva M. (2023). Complex I Inhibitor of Oxidative
Phosphorylation in
Advanced Solid Tumors and Acute Myeloid Leukemia: Phase I Trials. Nat. Med..

[14] Zhang X., Dang C. V. (2023). Time to Hit Pause on Mitochondria-Targeting
Cancer
Therapies. Nat. Med..

[15] Arrillaga-Romany I., Gardner S. L., Odia Y., Aguilera D., Allen J. E., Batchelor T., Butowski N., Chen C., Cloughesy T., Cluster A., de Groot J., Dixit K. S., Graber J. J., Haggiagi A. M., Harrison R. A., Kheradpour A., Kilburn L. B., Kurz S. C., Lu G., MacDonald T. J., Mehta M., Melemed A. S., Nghiemphu P. L., Ramage S. C., Shonka N., Sumrall A., Tarapore R. S., Taylor L., Umemura Y., Wen P. Y. (2024). ONC201 (Dordaviprone)
in Recurrent H3 K27M–Mutant Diffuse Midline Glioma. J. Clin. Oncol..

[16] Thompson W. R., Manuel R., Abbruscato A., Carr J., Campbell J., Hornby B., Vaz F. M., Vernon H. J. (2024). Long-Term Efficacy
and Safety of Elamipretide in Patients with Barth Syndrome: 168-Week
Open-Label Extension Results of TAZPOWER. Genet.
Med..

[17] Wang D., Wang W., Fang L., Qi L., Zhang Y., Liu J., Liang Y., Yang H., Wang M., Wei X., Jiang R., Liu Y., Zhou W., Fang X. (2023). Mitochondrial Protease Targeting
Chimeras for Mitochondrial Matrix Protein Degradation. J. Am. Chem. Soc..

[18] Murray K. O., Berryman-Maciel M., Darvish S., Coppock M. E., You Z., Chonchol M., Seals D. R., Rossman M. J. (2022). Mitochondrial-Targeted
Antioxidant Supplementation for Improving Age-Related Vascular Dysfunction
in Humans: A Study Protocol. Front. Physiol..

[19] Petrov A., Perekhvatova N., Skulachev M., Stein L., Ousler G. (2016). SkQ1 Ophthalmic
Solution for Dry Eye Treatment: Results of a Phase 2 Safety and Efficacy
Clinical Study in the Environment and During Challenge in the Controlled
Adverse Environment Model. Adv. Ther..

[20] Hayat U., Elliott G. T., Olszanski A. J., Altieri D. C. (2022). Feasibility and
Safety of Targeting Mitochondria for Cancer Therapy – Preclinical
Characterization of Gamitrinib, a First-in-Class, mitochondriaL-Targeted
Small Molecule Hsp90 Inhibitor. Cancer Biol.
Ther..

[21] Trendeleva T. A., Sukhanova E. I., Rogov A. G., Zvyagilskaya R. A., Seveina I. I., Ilyasova T. M., Cherepanov D. A., Skulachev V. P. (2013). Role
of Charge Screening and Delocalization for Lipophilic
Cation Permeability of Model and Mitochondrial Membranes. Mitochondrion.

[22] Benfeito S., Fernandes C., Chavarria D., Barreiro S., Cagide F., Sequeira L., Teixeira J., Silva R., Remião F., Oliveira P. J., Uriarte E., Borges F. (2023). Modulating Cytotoxicity
with Lego-like Chemistry: Upgrading Mitochondriotropic Antioxidants
with Prototypical Cationic Carrier Bricks. J.
Med. Chem..

[23] Bragadin M., Moret I., Piazza R., Grasso M., Manente S. (2002). The
Interactions of Cetyltrimethylammonium with Mitochondria:
An Uncoupler or a Detergent?. Chemosphere.

[24] Sukhanova E. I., Trendeleva T. A., Zvyagilskaya R. A. (2010). Interaction
of Yeast Mitochondria with Fatty Acids
and Mitochondria-Targeted Lipophilic Cations. Biochemistry.

[25] Antonenko Y. N., Khailova L. S., Knorre D. A., Markova O. V., Rokitskaya T. I., Ilyasova T. M., Severina I. I., Kotova E. A., Karavaeva Y. E., Prikhodko A. S., Severin F. F., Skulachev V. P. (2013). Penetrating
Cations Enhance Uncoupling Activity of Anionic Protonophores in Mitochondria. PLoS One.

[26] Trnka J., Elkalaf M., Anděl M. (2015). Lipophilic
Triphenylphosphonium Cations
Inhibit Mitochondrial Electron Transport Chain and Induce Mitochondrial
Proton Leak. PLoS One.

[27] Modica-Napolitano J.
S., Aprille J. R. (1987). Basis for
the Selective Cytotoxicity of Rhodamine 123. Cancer Res..

[28] Monzel A. S., Enríquez J. A., Picard M. (2023). Multifaceted Mitochondria: Moving
Mitochondrial Science beyond Function and Dysfunction. Nat. Metab..

[29] Zorova L. D., Popkov V. A., Plotnikov E. Y., Silachev D. N., Pevzner I. B., Jankauskas S. S., Babenko V. A., Zorov S. D., Balakireva A. V., Juhaszova M., Sollott S. J., Zorov D. B. (2018). Mitochondrial Membrane
Potential. Anal. Biochem..

[30] Murphy M. P. (1997). Selective
Targeting of Bioactive Compounds to Mitochondria. Trends Biotechnol..

[31] Kim S., Nam H. Y., Lee J., Seo J. (2020). Mitochondrion-Targeting
Peptides and Peptidomimetics: Recent Progress and Design Principles. Biochemistry.

[32] Skulachev V. P., Antonenko Y. N., Cherepanov D. A., Chernyak B. V., Izyumov D. S., Khailova L. S., Klishin S. S., Korshunova G. A., Lyamzaev K. G., Pletjushkina O. Y., Roginsky V. A., Rokitskaya T. I., Severin F. F., Severina I. I., Simonyan R. A., Skulachev M. V., Sumbatyan N. V., Sukhanova E. I., Tashlitsky V. N., Trendeleva T. A., Vyssokikh M. Y., Zvyagilskaya R. A. (2010). Prevention
of Cardiolipin Oxidation and Fatty Acid Cycling as Two Antioxidant
Mechanisms of Cationic Derivatives of Plastoquinone (SkQs). Biochim. Biophys. Acta, Bioenerg..

[33] Zielonka J., Joseph J., Sikora A., Hardy M., Ouari O., Vasquez-Vivar J., Cheng G., Lopez M., Kalyanaraman B. (2017). Mitochondria-Targeted
Triphenylphosphonium-Based Compounds: Syntheses, Mechanisms of Action,
and Therapeutic and Diagnostic Applications. Chem. Rev..

[34] Battogtokh G., Choi Y. S., Kang D. S., Park S. J., Shim M. S., Huh K. M., Cho Y.-Y., Lee J. Y., Lee H. S., Kang H. C. (2018). Mitochondria-Targeting Drug Conjugates
for Cytotoxic,
Anti-Oxidizing and Sensing Purposes: Current Strategies and Future
Perspectives. Acta Pharm. Sin. B.

[35] Qin X., Li H., Zhao H., Fang L., Wang X. (2024). Enhancing Healthy Aging
with Small Molecules: A Mitochondrial Perspective. Med. Res. Rev..

[36] Gubič Š., Hendrickx L. A., Toplak Ž., Sterle M., Peigneur S., Tomašič T., Pardo L. A., Tytgat J., Zega A., Mašič L.
P. (2021). Discovery of KV1.3 Ion
Channel Inhibitors: Medicinal Chemistry Approaches and Challenges. Med. Res. Rev..

[37] Pingitore V., Pancholi J., Hornsby T. W., Warne J., Pryce G., McCormick L. J., Hill J., Bhosale G., Peng J., Newton L. S., Towers G. J., Coles S. J., Chan A. W. E., Duchen M. R., Szabadkai G., Baker D., Selwood D. L. (2024). Delocalized
Quinolinium-Macrocyclic Peptides, an Atypical Chemotype for CNS Penetration. Sci. Adv..

[38] Ding Q., Wang X., Luo Y., Leng X., Li X., Gu M., Kim J. S. (2024). Mitochondria-Targeted
Fluorophore: State of the Art
and Future Trends. Coord. Chem. Rev..

[39] Jana R. D., Nguyen H. D., Yan G., Chen T.-Y., Do L. H. (2025). Reversing
Signs of Parkinsonism in a Cell Model Using Mitochondria-Targeted
Organoiridium Catalysis. J. Med. Chem..

[40] Dai N., Zhao H., Qi R., Chen Y., Lv F., Liu L., Wang S. (2020). Fluorescent
and Biocompatible Ruthenium-Coordinated
Oligo­(p-Phenylenevinylene) Nanocatalysts for Transfer Hydrogenation
in the Mitochondria of Living Cells. Chem. -
Eur. J..

[41] Kulkarni C. A., Fink B. D., Gibbs B. E., Chheda P. R., Wu M., Sivitz W. I., Kerns R. J. (2021). A Novel Triphenylphosphonium Carrier
to Target Mitochondria without Uncoupling Oxidative Phosphorylation. J. Med. Chem..

[42] Ong H. C., Coimbra J. T. S., Kwek G., Ramos M. J., Xing B., Fernandes P. A., García F. (2021). Alkyl vs. Aryl Modifications: A Comparative
Study on Modular Modifications of Triphenylphosphonium Mitochondrial
Vectors. RSC Chem. Biol..

[43] Hüser J., Rechenmacher C. E., Blatter L. A. (1998). Imaging the Permeability Pore Transition
in Single Mitochondria. Biophys. J..

[44] da
Cunha F. M., Torelli N. Q., Kowaltowski A. J. (2015). Mitochondrial
Retrograde Signaling: Triggers, Pathways, and Outcomes. Oxid. Med. Cell. Longevity.

[45] Suski, J. M. ; Lebiedzinska, M. ; Bonora, M. ; Pinton, P. ; Duszynski, J. ; Wieckowski, M. R. Relation Between Mitochondrial Membrane Potential and ROS Formation. In Mitochondrial Bioenergetics: Methods and Protocols; Palmeira, C. M. ; Moreno, A. J. , Eds.; Humana Press: Totowa, NJ, 2012; pp 183–205. 10.1007/978-1-61779-382-0_12 22057568

[46] Murphy M. P. (1989). Slip and
Leak in Mitochondrial Oxidative Phosphorylation. Biochim. Biophys. Acta, Bioenerg..

[47] Schenkel L. C., Bakovic M. (2014). Formation and Regulation
of Mitochondrial Membranes. Int. J. Cell Biol..

[48] Decker S. T., Funai K. (2024). Mitochondrial Membrane
Lipids in the Regulation of Bioenergetic Flux. Cell Metab..

[49] Liberman E. A., Skulachev V. P. (1970). Conversion
of Biomembrane-Produced Energy into Electric
Form. IV. General Discussion. Biochim. Biophys.
Acta, Bioenerg..

[50] Perry S. W., Norman J. P., Barbieri J., Brown E. B., Gelbard H. A. (2011). Mitochondrial
Membrane Potential Probes and the Proton Gradient: A Practical Usage
Guide. BioTechniques.

[51] Scaduto R. C., Grotyohann L. W. (1999). Measurement
of Mitochondrial Membrane Potential Using
Fluorescent Rhodamine Derivatives. Biophys.
J..

[52] Childress E. S., Alexopoulos S. J., Hoehn K. L., Santos W. L. (2018). Small Molecule
Mitochondrial
Uncouplers and Their Therapeutic Potential: Miniperspective. J. Med. Chem..

[53] Yoon N. G., Lee H., Kim S.-Y., Hu S., Kim D., Yang S., Hong K. B., Lee J. H., Kang S., Kim B.-G., Myung K., Lee C., Kang B. H. (2021). Mitoquinone
Inactivates Mitochondrial Chaperone TRAP1
by Blocking the Client Binding Site. J. Am.
Chem. Soc..

[54] Walther Nernst – Nobel Lecture. NobelPrize.org. https://www.nobelprize.org/prizes/chemistry/1920/nernst/lecture/ (accessed July 01, 2025).

[55] Zorova L.
D., Demchenko E. A., Korshunova G. A., Tashlitsky V. N., Zorov S. D., Andrianova N. V., Popkov V. A., Babenko V. A., Pevzner I. B., Silachev D. N., Plotnikov E. Y., Zorov D. B. (2022). Is the Mitochondrial Membrane Potential
(ΔΨ)
Correctly Assessed? Intracellular and Intramitochondrial Modifications
of the ΔΨ Probe, Rhodamine 123. Int. J. Mol. Sci..

[56] Grinius L. L., Jasaitis A. A., Kadziauskas Y. P., Liberman E. A., Skulachev V. P., Topali V. P., Tsofina L. M., Vladimirova M. A. (1970). Conversion
of Biomembrane-Produced Energy into Electric Form. I. Submitochondrial
Particles. Biochim. Biophys. Acta, Bioenerg..

[57] Bakeeva L. E., Grinius L. L., Jasaitis A. A., Kuliene V. V., Levitsky D. O., Liberman E. A., Severina I. I., Skulachev V. P. (1970). Conversion
of Biomembrane-Produced Energy into Electric Form. II. Intact Mitochondria. Biochim. Biophys. Acta, Bioenerg..

[58] Isaev P. I., Liberman E. A., Samuilov V. D., Skulachev V. P., Tsofina L. M. (1970). Conversion of Biomembrane-Produced
Energy into Electric
Form. III. Chromatophores of *Rhodospirillum rubrum*. Biochim. Biophys. Acta, Bioenerg..

[59] Skulachev V. P. (2005). How to
Clean the Dirtiest Place in the Cell: Cationic Antioxidants as Intramitochondrial
ROS Scavengers. IUBMB Life.

[60] Fantin V. R., Berardi M. J., Scorrano L., Korsmeyer S. J., Leder P. (2002). A Novel Mitochondriotoxic Small Molecule
That Selectively
Inhibits Tumor Cell Growth. Cancer Cell.

[61] Bernal S. D., Lampidis T. J., Summerhayes I. C., Chen L. B. (1982). Rhodamine-123 Selectively
Reduces Clonal Growth of Carcinoma Cells in Vitro. Science.

[62] Summerhayes I. C., Lampidis T. J., Bernal S. D., Nadakavukaren J. J., Nadakavukaren K. K., Shepherd E. L., Chen L. B. (1982). Unusual
Retention
of Rhodamine 123 by Mitochondria in Muscle and Carcinoma Cells. Proc. Natl. Acad. Sci. U.S.A..

[63] Nadakavukaren K. K., Nadakavukaren J. J., Chen L. B. (1985). Increased Rhodamine 123 Uptake by
Carcinoma Cells1. Cancer Res..

[64] Davis S., Weiss M. J., Wong J. R., Lampidis T. J., Chen L. B. (1985). Mitochondrial
and Plasma Membrane Potentials Cause Unusual Accumulation and Retention
of Rhodamine 123 by Human Breast Adenocarcinoma-Derived MCF-7 Cells. J. Biol. Chem..

[65] Zorova L. D., Abramicheva P. A., Andrianova N. V., Babenko V. A., Zorov S. D., Pevzner I. B., Popkov V. A., Semenovich D. S., Yakupova E. I., Silachev D. N., Plotnikov E. Y., Sukhikh G. T., Zorov D. B. (2024). Targeting Mitochondria
for Cancer
Treatment. Pharmaceutics.

[66] Houston M. A., Augenlicht L. H., Heerdt B. G. (2011). Stable Differences in Intrinsic Mitochondrial
Membrane Potential of Tumor Cell Subpopulations Reflect Phenotypic
Heterogeneity. Int. J. Cell Biol..

[67] Heerdt B. G., Houston M. A., Augenlicht L. H. (2005). The Intrinsic
Mitochondrial Membrane
Potential of Colonic Carcinoma Cells Is Linked to the Probability
of Tumor Progression. Cancer Res..

[68] Kuwahara Y., Tomita K., Roudkenar M. H., Roushandeh A. M., Urushihara Y., Igarashi K., Kurimasa A., Sato T. (2021). Decreased
Mitochondrial Membrane Potential Is an Indicator of Radioresistant
Cancer Cells. Life Sci..

[69] Momcilovic M., Jones A., Bailey S. T., Waldmann C. M., Li R., Lee J. T., Abdelhady G., Gomez A., Holloway T., Schmid E., Stout D., Fishbein M. C., Stiles L., Dabir D. V., Dubinett S. M., Christofk H., Shirihai O., Koehler C. M., Sadeghi S., Shackelford D. B. (2019). In Vivo
Imaging of Mitochondrial Membrane Potential in Non-Small-Cell Lung
Cancer. Nature.

[70] Forrest, M. D. Why Cancer Cells Have a More Hyperpolarised Mitochondrial Membrane Potential and Emergent Prospects for Therapy bioRxiv 2015 10.1101/025197.

[71] Begum H. M., Shen K. (2023). Intracellular and Microenvironmental Regulation of Mitochondrial
Membrane Potential in Cancer Cells. WIREs Mech.
Dis..

[72] Ong H. C., Coimbra J. T. S., Ramos M. J., Xing B., Fernandes P. A., García F. (2023). Beyond the TPP+ “Gold Standard”: A New
Generation Mitochondrial Delivery Vector Based on Extended PN Frameworks. Chem. Sci..

[73] Wang X., Ohlin C. A., Lu Q., Fei Z., Hu J., Dyson P. J. (2007). Cytotoxicity of Ionic Liquids and
Precursor Compounds
towards Human Cell Line HeLa. Green Chem..

[74] Millard M., Pathania D., Shabaik Y., Taheri L., Deng J., Neamati N. (2010). Preclinical Evaluation
of Novel Triphenylphosphonium
Salts with Broad-Spectrum Activity. PLoS One.

[75] Andreeva O. V., Voloshina A. D., Lyubina A. P., Garifullin B. F., Sapunova A. S., Amerhanova S. K., Strobykina I. Y., Belenok M. G., Babaeva O. B., Saifina L. F., Semenov V. E., Kataev V. E. (2024). Acetylenyl Substituted Nucleic Bases and Their Triphenylphosphonium
(TPP) Conjugates. Unexpected Surge in Cytotoxicity. Bioorg. Chem..

[76] Roelofs B. A., Ge S. X., Studlack P. E., Polster B. M. (2015). Low Micromolar Concentrations
of the Superoxide Probe MitoSOX Uncouple Neural Mitochondria and Inhibit
Complex IV. Free Radicals Biol. Med..

[77] Sodano F., Rolando B., Spyrakis F., Failla M., Lazzarato L., Gazzano E., Riganti C., Fruttero R., Gasco A., Sortino S. (2018). Tuning the Hydrophobicity
of a Mitochondria-Targeted
NO Photodonor. ChemMedChem.

[78] Asin-Cayuela J., Manas A.-R. B., James A. M., Smith R. A. J., Murphy M. P. (2004). Fine-Tuning
the Hydrophobicity of a Mitochondria-Targeted Antioxidant. FEBS Lett..

[79] Khailova L. S., Nazarov P. A., Sumbatyan N. V., Korshunova G. A., Rokitskaya T. I., Dedukhova V. I., Antonenko Y. N., Skulachev V. P. (2015). Uncoupling and Toxic Action of Alkyltriphenylphosphonium
Cations on Mitochondria and the Bacterium Bacillus Subtilis as a Function
of Alkyl Chain Length. Biochemistry.

[80] Uno S., Harkiss A. H., Chowdhury R., Caldwell S. T., Prime T. A., James A. M., Gallagher B., Prudent J., Hartley R. C., Murphy M. P. (2023). Incorporating
a Polyethyleneglycol Linker to Enhance
the Hydrophilicity of Mitochondria-Targeted Triphenylphosphonium Constructs. ChemBioChem.

[81] Kim Y.-S., Yang C.-T., Wang J., Wang L., Li Z.-B., Chen X., Liu S. (2008). Effects of
Targeting Moiety, Linker,
Bifunctional Chelator, and Molecular Charge on Biological Properties
of 64Cu-Labeled Triphenylphosphonium Cations. J. Med. Chem..

[82] Lei E. K., Kelley S. O. (2017). Delivery and Release
of Small-Molecule Probes in Mitochondria
Using Traceless Linkers. J. Am. Chem. Soc..

[83] Bashore F. M., Foley C. A., Ong H. W., Rectenwald J. M., Hanley R. P., Norris-Drouin J. L., Cholensky S. H., Mills C. A., Pearce K. H., Herring L. E., Kireev D., Frye S. V., James L. I. (2023). PROTAC Linkerology Leads to an Optimized
Bivalent Chemical Degrader of Polycomb Repressive Complex 2 (PRC2)
Components. ACS Chem. Biol..

[84] Sosič I., Bricelj A., Steinebach C. (2022). E3 Ligase
Ligand Chemistries: From
Building Blocks to Protein Degraders. Chem.
Soc. Rev..

[85] Su Z., Xiao D., Xie F., Liu L., Wang Y., Fan S., Zhou X., Li S. (2021). Antibody–Drug Conjugates:
Recent Advances in Linker Chemistry. Acta Pharm.
Sin. B.

[86] Bryant K. G., Chae Y. C., Martinez R. L., Gordon J. C., Elokely K. M., Kossenkov A. V., Grant S., Childers W. E., Abou-Gharbia M., Altieri D. C. (2017). A Mitochondrial-Targeted Purine-Based
HSP90 Antagonist
for Leukemia Therapy. Oncotarget.

[87] Qian K., Gao S., Jiang Z., Ding Q., Cheng Z. (2024). Recent Advances in
Mitochondria-Targeting Theranostic Agents. Exploration.

[88] Lv W., Zhang Z., Zhang K. Y., Yang H., Liu S., Xu A., Guo S., Zhao Q., Huang W. (2016). A Mitochondria-Targeted
Photosensitizer Showing Improved Photodynamic Therapy Effects Under
Hypoxia. Angew. Chem., Int. Ed..

[89] Kang B. H., Plescia J., Song H. Y., Meli M., Colombo G., Beebe K., Scroggins B., Neckers L., Altieri D. C. (2009). Combinatorial
Drug Design Targeting Multiple Cancer
Signaling Networks Controlled by Mitochondrial Hsp90. J. Clin. Invest..

[90] Kang B. H., Plescia J., Dohi T., Rosa J., Doxsey S. J., Altieri D. C. (2007). Regulation of Tumor
Cell Mitochondrial Homeostasis
by an Organelle-Specific Hsp90 Chaperone Network. Cell.

[91] Al
Tahan M. A., Al Tahan S. (2024). Pioneering Advances and Innovative
Applications of Mesoporous Carriers for Mitochondria-Targeted Therapeutics. Br. J. Biomed. Sci..

[92] Guo X., Yang N., Ji W., Zhang H., Dong X., Zhou Z., Li L., Shen H.-M., Yao S. Q., Huang W. (2021). Mito-Bomb: Targeting Mitochondria for Cancer Therapy. Adv. Mater..

[93] Malouitre S., Dube H., Selwood D., Crompton M. (2010). Mitochondrial Targeting
of Cyclosporin A Enables Selective Inhibition of Cyclophilin-D and
Enhanced Cytoprotection after Glucose and Oxygen Deprivation. Biochem. J..

[94] Yang S., Yoon N. G., Park M.-A., Yun J., Im J. Y., Kang B. H., Kang S. (2022). Triphenylphosphonium Conjugation
to a TRAP1 Inhibitor, 2-Amino-6-Chloro-7,9-Dihydro-8H-Purin-8-One
Increases Antiproliferative Activity. Bioorg.
Chem..

[95] Leanza L., Romio M., Becker K. A., Azzolini M., Trentin L., Managò A., Venturini E., Zaccagnino A., Mattarei A., Carraretto L., Urbani A., Kadow S., Biasutto L., Martini V., Severin F., Peruzzo R., Trimarco V., Egberts J.-H., Hauser C., Visentin A., Semenzato G., Kalthoff H., Zoratti M., Gulbins E., Paradisi C., Szabo I. (2017). Direct Pharmacological Targeting
of a Mitochondrial Ion Channel Selectively Kills Tumor Cells In Vivo. Cancer Cell.

[96] Yoo I., Ahn I., Lee J., Lee N. (2024). Extracellular
Flux Assay (Seahorse Assay): Diverse
Applications in Metabolic Research across Biological Disciplines. Mol. Cells.

[97] Sivandzade F., Bhalerao A., Cucullo L. (2019). Analysis of the Mitochondrial
Membrane
Potential Using the Cationic JC-1 Dye as a Sensitive Fluorescent Probe. Bio-Protoc..

[98] Ma C., Xia F., Kelley S. O. (2020). Mitochondrial Targeting of Probes and Therapeutics
to the Powerhouse of the Cell. Bioconjugate
Chem..

[99] Li W., Wilson G. C., Bachmann M., Wang J., Mattarei A., Paradisi C., Edwards M. J., Szabo I., Gulbins E., Ahmad S. A., Patel S. H. (2022). Inhibition
of a Mitochondrial Potassium
Channel in Combination with Gemcitabine and Abraxane Drastically Reduces
Pancreatic Ductal Adenocarcinoma in an Immunocompetent Orthotopic
Murine Model. Cancers.

[100] Karwi Q. G., Bornbaum J., Boengler K., Torregrossa R., Whiteman M., Wood M. E., Schulz R., Baxter G. F. (2017). AP39, a
Mitochondria-Targeting Hydrogen Sulfide (H_2_S) Donor, Protects
against Myocardial Reperfusion Injury Independently of Salvage Kinase
Signalling: Cardioprotection with AP39. Br.
J. Pharmacol..

[101] Marder L. S., Lunardi J., Renard G., Rostirolla D. C., Petersen G. O., Nunes J. E. S., de Souza A. P. D., de
O Dias A. C., Chies J. M., Basso L. A., Santos D. S., Bizarro C. V. (2014). Production of Recombinant Human Annexin V by Fed-Batch
Cultivation. BMC Biotechnol..

[102] Marsh, W. Propidium. In xPharm: The Comprehensive Pharmacology Reference; Enna, S. J. ; Bylund, D. B. , Eds.; Elsevier: New York, 2007; pp 1–2.

[103] Costigan A., Hollville E., Martin S. J. (2023). Discriminating Between
Apoptosis, Necrosis, Necroptosis, and Ferroptosis by Microscopy and
Flow Cytometry. Curr. Protoc..

[104] Riss, T. L. ; Moravec, R. A. ; Niles, A. L. ; Duellman, S. ; Benink, H. A. ; Worzella, T. J. ; Minor, L. Cell Viability Assays. In Assay Guidance Manual; Markossian, S. ; Grossman, A. ; Baskir, H. ; Arkin, M. ; Auld, D. ; Austin, C. ; Baell, J. ; Brimacombe, K. ; Chung, T. D. Y. ; Coussens, N. P. ; Dahlin, J. L. ; Devanarayan, V. ; Foley, T. L. ; Glicksman, M. ; Gorshkov, K. ; Grotegut, S. ; Hall, M. D. ; Hoare, S. ; Inglese, J. ; Iversen, P. W. ; Lal-Nag, M. ; Li, Z. ; Manro, J. R. ; McGee, J. ; Norvil, A. ; Pearson, M. ; Riss, T. ; Saradjian, P. ; Sittampalam, G. S. ; Tarselli, M. A. ; Trask, O. J. ; Weidner, J. R. ; Wildey, M. J. ; Wilson, K. ; Xia, M. ; Xu, X. , Eds.; Eli Lilly & Company and the National Center for Advancing Translational Sciences: Bethesda, MD, 2004.

[105] Assay Guidance Manual; Markossian, S. ; Grossman, A. ; Baskir, H. ; Arkin, M. ; Auld, D. ; Austin, C. ; Baell, J. ; Brimacombe, K. ; Chung, T. D. Y. ; Coussens, N. P. ; Dahlin, J. L. ; Devanarayan, V. ; Foley, T. L. ; Glicksman, M. ; Gorshkov, K. ; Grotegut, S. ; Hall, M. D. ; Hoare, S. ; Inglese, J. ; Iversen, P. W. ; Lal-Nag, M. ; Li, Z. ; Manro, J. R. ; McGee, J. ; Norvil, A. ; Pearson, M. ; Riss, T. ; Saradjian, P. ; Sittampalam, G. S. ; Tarselli, M. A. ; Trask, O. J. ; Weidner, J. R. ; Wildey, M. J. ; Wilson, K. ; Xia, M. ; Xu, X. , Eds.; Eli Lilly & Company and the National Center for Advancing Translational Sciences: Bethesda, MD, 2004. 22553861

[106] Desousa B. R., Kim K. K., Jones A. E., Ball A. B., Hsieh W. Y., Swain P., Morrow D. H., Brownstein A. J., Ferrick D. A., Shirihai O. S., Neilson A., Nathanson D. A., Rogers G. W., Dranka B. P., Murphy A. N., Affourtit C., Bensinger S. J., Stiles L., Romero N., Divakaruni A. S. (2023). Calculation
of ATP Production Rates Using the Seahorse XF Analyzer. EMBO Rep..

[107] Hanson K. M., Finkelstein J. N. (2019). An Accessible and High-Throughput
Strategy of Continuously Monitoring Apoptosis by Fluorescent Detection
of Caspase Activation. Anal. Biochem..

[108] Modica-Napolitano J.
S., Aprille J. R. (2001). Delocalized
Lipophilic Cations Selectively
Target the Mitochondria of Carcinoma Cells. Adv. Drug Delivery Rev..

[109] Walter E. R. H., Lee L. C.-C., Leung P. K.-K., Lo K. K.-W., Long N. J. (2024). Mitochondria-Targeting Biocompatible
Fluorescent BODIPY
Probes. Chem. Sci..

[110] Rokitskaya T. I., Kotova E. A., Luzhkov V. B., Kirsanov R. S., Aleksandrova E. V., Korshunova G. A., Tashlitsky V. N., Antonenko Y. N. (2021). Lipophilic Ion Aromaticity Is Not
Important for Permeability
across Lipid Membranes. Biochim. Biophys. Acta,
Biomembr..

[111] Young R. J., Green D. V. S., Luscombe C. N., Hill A. P. (2011). Getting
Physical in Drug Discovery II: The Impact of Chromatographic Hydrophobicity
Measurements and Aromaticity. Drug Discovery
Today.

[112] Ju W., Li N., Wang J., Yu N., Lei Z., Zhang L., Sun J., Chen L. (2021). Design and Synthesis
of Novel Mitochondria-Targeted CDDO Derivatives as Potential Anti-Cancer
Agents. Bioorg. Chem..

[113] Millard M., Gallagher J. D., Olenyuk B. Z., Neamati N. (2013). A Selective
Mitochondrial-Targeted Chlorambucil with Remarkable Cytotoxicity in
Breast and Pancreatic Cancers. J. Med. Chem..

[114] Pantelia A., Daskalaki I., Cuquerella M. C., Rotas G., Miranda M. A., Vougioukalakis G. C. (2019). Synthesis
and Chemiluminescent Properties of Amino-Acylated Luminol Derivatives
Bearing Phosphonium Cations. Molecules.

[115] Daina A., Michielin O., Zoete V. (2017). SwissADME: A Free Web
Tool to Evaluate Pharmacokinetics, Drug-Likeness and Medicinal Chemistry
Friendliness of Small Molecules. Sci. Rep..

[116] Daina A., Michielin O., Zoete V. (2014). iLOGP: A Simple, Robust,
and Efficient Description of n-Octanol/Water Partition Coefficient
for Drug Design Using the GB/SA Approach. J.
Chem. Inf. Model..

[117] Haslop A., Wells L., Gee A., Plisson C., Long N. (2014). One-Pot Multi-Tracer Synthesis of
Novel 18F-Labeled PET Imaging Agents. Mol. Pharmaceutics.

[118] Smith A.
J., Osborne B. E., Keeling G. P., Blower P. J., Southworth R., Long N. J. (2020). DO2A-Based Ligands for Gallium-68
Chelation: Synthesis, Radiochemistry and Ex Vivo Cardiac Uptake. Dalton Trans..

[119] Ong H. C., Hu Z., Coimbra J. T. S., Ramos M. J., Kon O. L., Xing B., Yeow E. K. L., Fernandes P. A., García F. (2019). Enabling Mitochondrial Uptake of
Lipophilic Dications
Using Methylated Triphenylphosphonium Moieties. Inorg. Chem..

[120] Hu Z., Sim Y., Kon O. L., Ng W. H., Ribeiro A. J. M., Ramos M. J., Fernandes P. A., Ganguly R., Xing B., García F., Yeow E. K. L. (2017). Unique Triphenylphosphonium Derivatives
for Enhanced Mitochondrial Uptake and Photodynamic Therapy. Bioconjugate Chem..

[121] Gruenwald H. K., Kerns R. J. (2022). Stability of Phenyl-Modified
Triphenylphosphonium
Conjugates and Interactions with DTPA. ACS Omega.

[122] AbuEid M., Keyes R. F., McAllister D., Peterson F., Kadamberi I. P., Sprague D. J., Chaluvally-Raghavan P., Smith B. C., Dwinell M. B. (2022). Fluorinated
Triphenylphosphonium
Analogs Improve Cell Selectivity and *in Vivo* Detection
of Mito-Metformin. iScience.

[123] Dube H., Selwood D., Malouitre S., Capano M., Simone M. I., Crompton M. (2012). A Mitochondrial-Targeted
Cyclosporin A with High Binding Affinity for Cyclophilin D Yields
Improved Cytoprotection of Cardiomyocytes. Biochem.
J..

[124] Warne J., Pryce G., Hill J. M., Shi X., Lennerås F., Puentes F., Kip M., Hilditch L., Walker P., Simone M. I., Chan A. W. E., Towers G. J., Coker A. R., Duchen M. R., Szabadkai G., Baker D., Selwood D. L. (2016). Selective
Inhibition of the Mitochondrial
Permeability Transition Pore Protects against Neurodegeneration in
Experimental Multiple Sclerosis*. J. Biol. Chem..

