# Supplementary material for: Lipophilic Cations as Mitochondria-Targeting Moieties: Recent Progress and Design Principles for Medicinal Chemistry
Source: J Med Chem. 2025 Nov 17;68(22):23690–704. doi: 10.1021/acs.jmedchem.5c02076 (PMC12670399; doi:10.1021/acs.jmedchem.5c02076)
Supplement: Supplementary file 1 [file jm5c02076_si_001.pdf]

Lipophilic Cations as Mitochondria-Targeting Moieties: Recent Progress and Design Principles for Medicinal Chemistry

Ivan Džajić,[†] Tihomir Tomašič,[†] Luis A. Pardo,[‡] Lucija Peterlin Mašič,[†] Andrej Emanuel Cotman[†,*]

[†]University of Ljubljana, Faculty of Pharmacy, Aškerčeva cesta 7, 1000 Ljubljana, Slovenia.

[‡]Max Planck Institute for Multidisciplinary Sciences, City Campus, Herrmann-Rein-Str. 3, 37075 Göttingen, Germany

* Email: andrej.emanuel.cotman@ffa.uni-lj.si

Supporting information

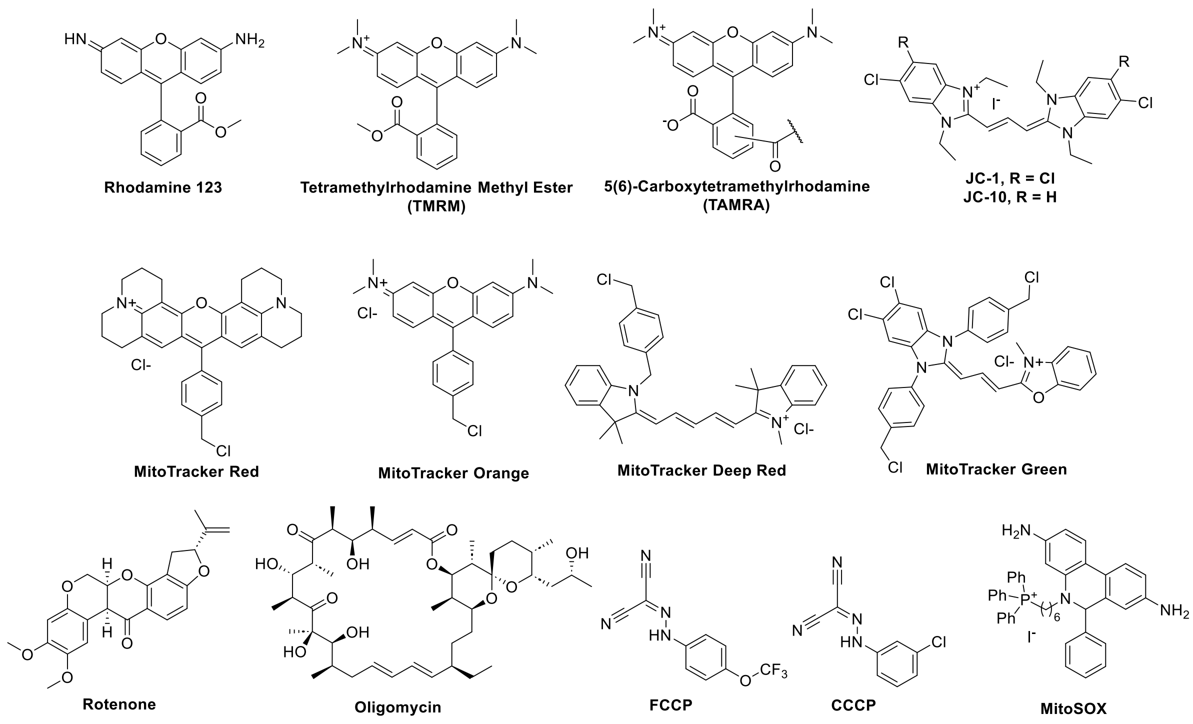

Figure S1. Structures of some fluorescent dyes and reagents used in mitochondrial research.
